# Machine learning-guided analysis of metabolomic alterations in Parkinson’s disease with comorbid symptoms

**DOI:** 10.3389/fnagi.2025.1753016

**Published:** 2026-01-13

**Authors:** Ran Sun, Lin Wang, Yanli Wang, Jinghui Feng, Xingrao Wu, Jinbiao Li, Meng Wang, Wenxuan Chen, Hongping Lai, Hao Wang, Yong Xia

**Affiliations:** 1 Department of Neurology, Affiliated Hospital of Jining Medical University, Jining, China; 2 Clinical Medical College, Jining Medical University, Jining, China; 3 Jining Key Laboratory of Collaborative Innovation and Translation in Medicine, Engineering, and Pharmaceuticals, School of Pharmaceutical Engineering, Jining Medical University, Jining, China

**Keywords:** disease diagnosis, LC-MS/MS, machine learning, metabolomic, Parkinson’s disease

## Abstract

**Introduction:**

As a common neurodegenerative disorder, Parkinson’s disease (PD) primarily affects dopaminergic neurons, leading to progressive motor disabilities along with a spectrum of non-motor complications. The early identification of Parkinson’s disease, as well as the exploration of biomarkers related to its associated comorbidities, remains an important focus of current research.

**Methods:**

In this study, a metabolomics approach combined with machine learning techniques was applied to explore potential biomarkers for PD and its related comorbid conditions. Using liquid chromatography–tandem mass spectrometry (LC–MS/MS), blood plasma samples were analyzed from individuals with PD, PD with rapid eye movement sleep behavior disorder (PD+RBD), PD with insomnia (PD + insomnia), and healthy controls, resulting in the detection of 2,601 metabolites. Multivariate statistical methods—including the unsupervised principal component analysis (PCA) and the supervised techniques of partial least squares discriminant analysis (PLS-DA) and orthogonal partial least squares discriminant analysis (OPLS-DA)—were employed to investigate intergroup metabolic variations. Machine learning algorithms, such as recursive feature elimination in conjunction with logistic regression, random forest, and support vector machines, were used to assist in selecting discriminative metabolites and constructing classification models.

**Results:**

These models showed strong internal performance in distinguishing PD from healthy individuals and in characterizing PD patients with non-motor comorbidities such as RBD and insomnia. Overall, the results suggest that metabolic biomarkers may provide valuable insights into disease-related and symptom-associated metabolic alterations in Parkinson’s disease.

**Discussion:**

This study provides a basis for future investigations aimed at validating these findings and further exploring their potential relevance in clinical research.

## 1 Introduction

Parkinson’s disease (PD) is a progressive neurological condition primarily impacting the central nervous system. It involves the gradual loss of dopaminergic neurons within the substantia nigra, which results in classical motor impairments, including tremor, muscle stiffness, slowed movement, and postural imbalance (Bloem et al., 2021; Morris et al., 2024; Weintraub et al., 2022). However, PD’s impact extends beyond motor dysfunctions. Non-motor symptoms, including rapid eye movement sleep behavior disorder (RBD), insomnia, autonomic dysfunction, depression, anxiety, and cognitive decline, often precede the onset of motor symptoms (Si et al., 2022; Huang et al., 2023; Mizrahi-Kliger et al., 2022). These non-motor manifestations provide important insights into the early stages of PD, suggesting their potential as biomarkers for early diagnosis, disease monitoring, and progression tracking (Qu et al., 2023; Liu et al., 2022; Zarkali et al., 2024). The global prevalence of PD is increasing rapidly, with over 6 million people currently affected, and this number is expected to rise due to the aging population. This trend highlights the urgent need for more efficient diagnostic tools and therapeutic interventions (Su et al., 2025; Bloem et al., 2021; Schiess et al., 2022).

Despite significant progress in PD research, clinical diagnosis still heavily relies on subjective evaluations of motor symptoms, which can vary widely between clinicians (Silva et al., 2023; Church, 2021; Antonini et al., 2023; Alharbi et al., 2024). This variability contributes to a high rate of misdiagnosis or missed diagnosis. Although imaging techniques and genetic testing offer more objective diagnostic options, their high costs and limited accessibility hinder their widespread use in clinical settings (Tolosa et al., 2021; Droby et al., 2023; Bidesi et al., 2021; Pal et al., 2023; Zhang, 2022; Bi et al., 2021). In recent years, metabolomics has gained attention as a promising method to explore the underlying pathophysiology of PD and to identify potential biomarkers (Li et al., 2022; de Lope et al., 2024; Santos et al., 2024; Paul et al., 2023). Advanced analytical techniques—such as High-Performance Liquid Chromatography and mass spectrometry—enable the investigation of metabolites present in biological specimens like blood and cerebrospinal fluid. This metabolic profiling offers valuable insights into Parkinson’s Disease and supports early diagnosis, disease monitoring, and patient classification (Santos-Reboucas et al., 2023; Brzenczek et al., 2024; Dahabiyeh et al., 2024; Gonzalez-Riano et al., 2021).

However, metabolomics data are typically high-dimensional, with small sample sizes and substantial noise, which pose significant challenges for traditional statistical methods (Galal et al., 2022; Taheri et al., 2025; Gong et al., 2023). Recently, machine learning (ML) techniques have been integrated into metabolomics research as a powerful approach to address these challenges (Xu et al., 2024; Sanches et al., 2024; Bissan et al., 2025; Elguoshy et al., 2025). By applying ML algorithms, complex, high-dimensional datasets can be analyzed to uncover hidden patterns, resulting in more accurate predictions, better feature selection, and a deeper understanding of disease biomarkers (Zhang et al., 2023; Shang et al., 2024). Machine learning has already achieved considerable success in the study of other neurological disorders, such as Alzheimer’s disease and multiple sclerosis (Arya et al., 2023; Grueso and Viejo-Sobera, 2021; Zhao et al., 2023; Diogo et al., 2022). In PD research, ML methods have shown potential not only in distinguishing PD patients from healthy controls, but also in identifying shifts in metabolic pathways linked to different clinical subtypes. These advancements lay the groundwork for future mechanistic studies and open the door to personalized medicine and targeted therapeutic strategies (Helaly et al., 2022; Cheung et al., 2022; Arafa et al., 2024).

This study utilized Liquid Chromatography–Tandem Mass Spectrometry to examine metabolic differences across four cohorts: PD, PD accompanied by Rapid Eye Movement Sleep Behavior Disorder, PD with insomnia, and healthy individuals. A total of 2,601 metabolites were identified, and subsequent statistical analysis revealed significant differences across the groups. Principal Component Analysis (PCA), Partial Least Squares Discriminant Analysis (PLS-DA), and Orthogonal Partial Least Squares Discriminant Analysis (OPLS-DA) were used to explore metabolic variations, refine the analysis, and identify key metabolites. Machine learning techniques, particularly Recursive Feature Elimination (RFE), were integrated with algorithms such as Logistic Regression, Random Forest, and Support Vector Machine (SVM) to identify the most discriminative metabolites. By incorporating these key metabolites into predictive models, we significantly improved classification accuracy in distinguishing PD from non-PD individuals, as well as identifying distinct clinical manifestations of PD, such as PD + insomnia vs. PD and PD + RBD vs. PD + insomnia. This iterative optimization process enabled the identification of robust and reliable biomarkers, which show promising clinical potential for early diagnosis, classification of diverse clinical manifestations, and continuous monitoring of Parkinson’s Disease progression.

## 2 Materials and methods

### 2.1 Reagents

For this metabolomic analysis, reagents were selected to the highest standards to ensure precision and accuracy during sample preparation and analysis. Key reagents included high-purity solvents such as methanol (A452-4, Fisher Scientific, HPLC grade, 99.9%) and acetonitrile (A998-4, Fisher Scientific, HPLC grade, 99.95%), essential for protein precipitation and metabolite extraction. Formic acid (A117-50, Fisher Scientific, HPLC grade, 99.0%) was used to modify the mobile phase and enhance ionization efficiency during mass spectrometry. Ultrapure water (Wahaha) was incorporated in the mobile phase. Internal standards, including L-2-chlorophenylalanine (C2001, Shanghai Hengchuang Bio, 98.0%), succinic-d4 (293075-1G, Sigma, 98.0%), L-valine-d8 (HY-I1124, Shanghai Haoyuan Bio, 98.0%), and cholic acid-D4 (S22155-50 mg, Shanghai Yuanye Bio, 98.0%), were added to ensure accurate quantification and normalization. These high-quality reagents were chosen for their compatibility with the LC–MS system, ensuring minimal interference during analysis and guaranteeing precise, reproducible results.

### 2.2 Instruments

Metabolite profiling was carried out via liquid chromatography-tandem mass spectrometry, utilizing the Waters ACQUITY UPLC I-Class Plus system combined with a Thermo QE high-resolution mass spectrometer. Separation was achieved using an AC-QUITY UPLC HSS T3 column (100 mm × 2.1 mm, 1.8 μm), maintained at 45 °C. The mobile phase included 0.1% formic acid in water (solvent A) and acetonitrile (solvent B), delivered at a constant flow rate of 0.35 mL/min. A programmed gradient elution was applied, transitioning from 95% A at 0 min to 0% A at 14 min, with intermediate steps at 2, 4, 8, and 10 min. The system returned to baseline conditions at 15 min for re-equilibration. Mass spectrometric detection was performed under dual ionization modes with a spray voltage of 3,800 V (positive) and −3,200 V (negative), a capillary temperature of 320 °C, a sheath gas flow rate of 35 arb, and an auxiliary gas flow rate of 8 arb. The instrument operated across an m/z range of 70 to 1,050, with resolution settings of 70,000 (MS) and 17,500 (MS/MS). Full scan acquisition captured both parent and fragment ions for each compound. Raw data were analyzed using Progenesis QI v3.0 for peak picking, alignment, and integration, followed by normalization, imputation, and transformation for downstream statistical analysis.

### 2.3 Sample collection

In this study, peripheral blood samples were collected from individuals diagnosed with Parkinson’s disease (PD), PD accompanied by Rapid Eye Movement Sleep Behavior Disorder (PD + RBD), PD with insomnia (PD + insomnia), and from healthy volunteers (HC), all recruited at the Affiliated Hospital of Jining Medical University under ethical approval (review number: 2023-07-C005). Standard venipuncture procedures were used to draw approximately 10 mL of blood, which was then transferred into EDTA-containing vacutainer tubes. After centrifugation, plasma was separated and preserved at −80 °C for later metabolomics profiling. The sample groups were as follows: 20 plasma samples from healthy individuals served as the control group; 31 samples were derived from patients with PD only; 23 were from patients diagnosed with both PD and insomnia; and 37 were from patients presenting with both PD and RBD. Comprehensive clinical information was collected for all Parkinson’s disease (PD) patients, including age, sex, disease duration, Hoehn and Yahr (H–Y) stage, Unified Parkinson’s Disease Rating Scale (UPDRS) scores, and medication history. These variables were used to characterize disease severity and clinical heterogeneity across study groups. Insomnia was assessed using the Athens Insomnia Scale (AIS), a validated self-report questionnaire. Participants with AIS total scores exceeding the established diagnostic threshold were classified as having insomnia. Rapid eye movement sleep behavior disorder (RBD) was evaluated using the REM Sleep Behavior Disorder Questionnaire–Hong Kong (RBDQ-HK), which captures both lifetime and recent RBD-related behaviors. To reduce potential metabolic confounding, patients with conditions known to substantially affect systemic metabolism, including diabetes mellitus, thyroid disease, obesity, gout, or severe systemic infection, were excluded. PD patients with sleep disorders other than insomnia or RBD were not included in the present study. The inclusion of these well-defined and diverse subject groups enabled a systematic analysis of metabolic perturbations linked to Parkinson’s disease and its associated non-motor conditions, laying the groundwork for comprehensive metabolomic investigations.

### 2.4 Liquid chromatography–tandem mass spectrometry analysis and data preprocessing

Metabolomic profiling in this study utilized tandem liquid chromatography-mass spectrometry (LC–MS/MS), conducted on a Waters ACQUITY UPLC I-Class Plus system, which was interfaced with a Thermo QE high-resolution mass analyzer. Chromatographic separation was performed using an ACQUITY UPLC HSS T3 column (100 mm × 2.1 mm, 1.8 μm) maintained at 45 °C. The mobile phase consisted of two eluents: (A) ultrapure water containing 0.1% formic acid and (B) acetonitrile. The gradient program followed these steps: initial composition of 95% A and 5% B at 0 min remained unchanged until 2 min, then shifted to 70% A/30% B at 4 min, 50% A/50% B at 8 min, 20% A/80% B at 10 min, and finally 0% A/100% B at 14 min, before returning to starting conditions by 15 min for system re-equilibration. The mass spectrometer was configured to alternate between positive and negative ion detection modes. Ionization voltages were set at +3,800 V and −3,200 V, respectively. Additional parameters included a capillary temperature of 320 °C, a sheath gas flow of 35 arbitrary units (arb), and auxiliary gas flow at 8 arb. The mass scan range was defined from m/z 70 to 1,050. Resolution settings were 70,000 for MS1 (full scan) and 17,500 for MS/MS (fragmentation). Full scan acquisition was employed to simultaneously record both precursor and product ion data for comprehensive metabolite identification. Data processing was conducted using Progenesis QI version 3.0, enabling peak extraction, alignment, and integration. Subsequent preprocessing steps—such as signal normalization, batch effect adjustment, baseline correction, and retention time calibration—were applied to enhance inter-sample comparability. Missing values were imputed where necessary, and all intensity data were log2-transformed prior to downstream statistical analyses. To monitor analytical stability during LC–MS/MS data acquisition, pooled quality control (QC) samples were prepared and injected throughout the analytical sequence. Quality control procedures included principal component analysis (PCA) of QC samples, hierarchical clustering analysis, QC correlation assessment, and inspection of metabolite intensity distributions. During data preprocessing, ion features were filtered based on relative standard deviation (RSD) calculated from QC samples. Features with RSD values greater than 30% were excluded, as RSD is equivalent to the coefficient of variation (CV) and serves as a quantitative measure of technical variability.

### 2.5 Statistical analysis and multivariate analysis

Given the high-dimensional nature of untargeted metabolomics data (p ≫ n), multivariate analyses were conducted within an exploratory and hypothesis-generating framework rather than for definitive classification purposes. Principal Component Analysis (PCA) was performed as an unsupervised method to reduce data dimensionality and to explore the overall variance structure among samples. PCA was primarily used to assess data quality, visualize global metabolic patterns, and identify potential outliers or trends across experimental groups (PD, PD + RBD, PD + insomnia, and healthy controls). PCA results were not used for inferential or predictive modeling. Partial Least Squares Discriminant Analysis (PLS-DA) was subsequently applied to visualize group-related metabolic patterns and to explore metabolites contributing to between-group variation. As a supervised method, PLS-DA constructs latent variables that maximize covariance between metabolite profiles and predefined group labels. In this study, PLS-DA was used to assist in pattern recognition and variable contribution assessment rather than to establish robust classification performance. Orthogonal Partial Least Squares Discriminant Analysis (OPLS-DA) was further employed to improve model interpretability by separating predictive variance associated with group differences from orthogonal variance unrelated to classification. This approach facilitated the identification of metabolites contributing to group separation while reducing noise; however, results were interpreted cautiously given the limited sample size and high dimensionality of the data.

### 2.6 Feature selection and model development

To improve both the classification accuracy and predictive strength of the metabolomic analysis, we adopted Recursive Feature Elimination (RFE) in conjunction with several machine learning techniques. RFE, through iterative elimination of less informative features, enables the selection of the most impactful variables for model training. To ensure selection stability across different algorithms, three models—Logistic Regression, Random Forest, and Support Vector Machine (SVM)—were independently applied for feature selection. The intersection of features derived from all three approaches yielded a consensus set of candidate metabolites for downstream analysis. Subsequently, a range of classification models, including K-Nearest Neighbors (KNN), Naive Bayes, Random Forest, SVM, and XGBoost, were trained to distinguish between multiple groups: PD versus healthy controls, PD with insomnia versus PD alone, and PD + RBD versus PD + insomnia. Binary labels were encoded using LabelEncoder, and data were split into training and testing partitions using a stratified hold-out strategy (train_test_split, test size = 0.30, random state = 42). For distance- and kernel-based models (KNN and SVM), z-score standardization was applied using StandardScaler within a scikit-learn Pipeline, where the scaler was fit on the training data only and then applied to the test set. Random Forest and Naive Bayes were trained on the original feature scale. Model performance was evaluated on both training and testing partitions using accuracy, sensitivity, specificity, PPV, NPV, F1 score, and AUC. ROC curves were generated for each model, and 95% confidence intervals for AUC were estimated using bootstrap resampling (n = 2000). Hyperparameters were specified *a priori* and were not optimized via systematic cross-validated tuning. Nested cross-validation and external validation were not performed; therefore, performance metrics should be interpreted as exploratory estimates reflecting internal separability within the dataset. The performance comparison identified Random Forest, SVM, and XGBoost as the top-performing classifiers, exhibiting high internal performance and strong separation within the analyzed dataset. These algorithms were further used to rank metabolite importance, supporting the construction of a final predictive model leveraging the most discriminative biomarkers. This integrative approach enhances both diagnostic accuracy and the clinical relevance of the metabolomic signatures.

## 3 Results

### 3.1 LC–MS analysis and differential metabolite identification

**Figure 1:**
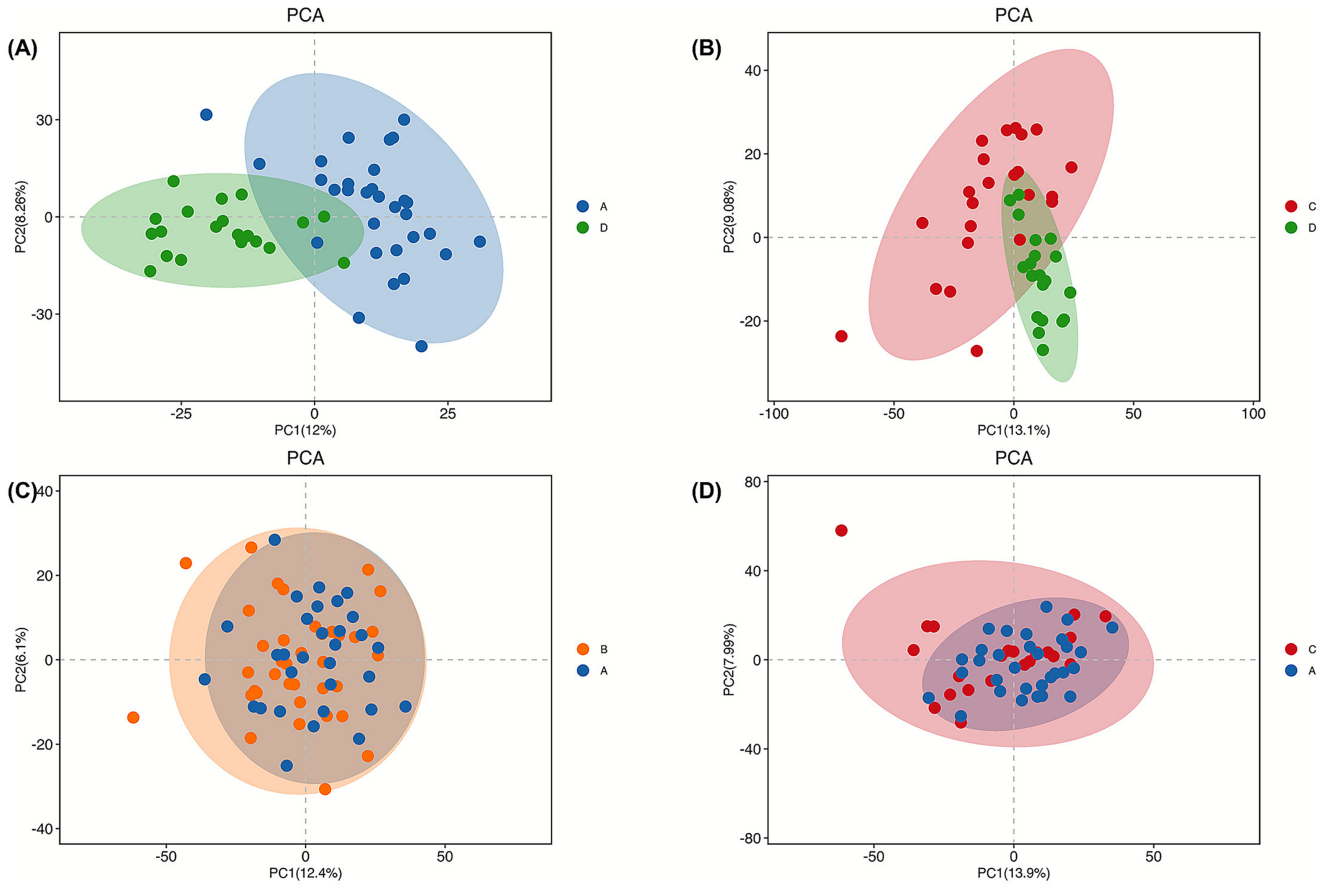
PCA score plot. **(A)** PCA score plot for the separation between PD and HC. **(B)** PCA score plot for the separation between PD and PD + insomnia. **(C)** PCA score plot for the separation between PD and PD + RBD. **(D)** PCA score plot for the separation between PD and PD + insomnia.

**Figure 2:**
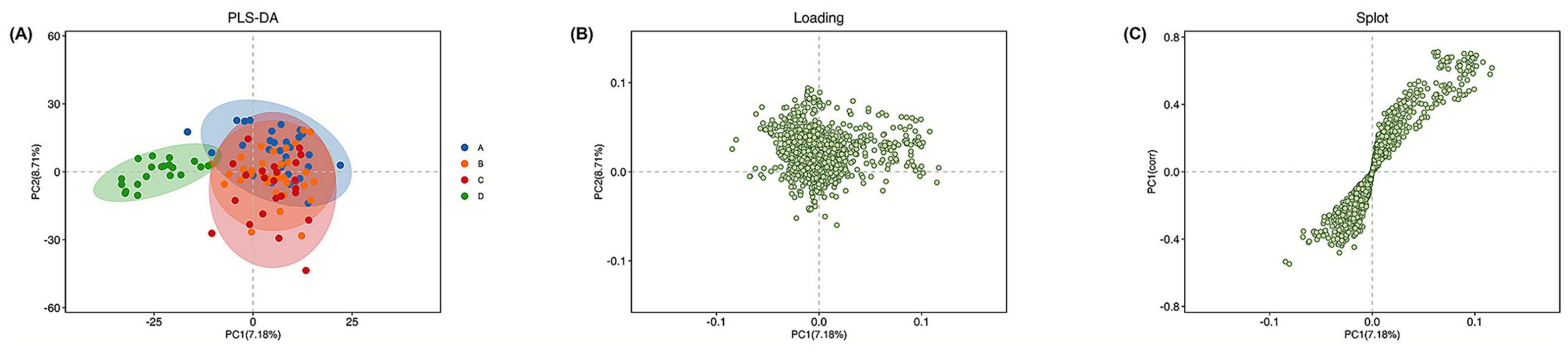
PLS-DA analysis. **(A)** PLS-DA score plot of PD, PD + RBD, PD + insomnia, and healthy controls based on their metabolic profiles. **(B)** The contribution of each metabolite to the separation between groups in the PLS-DA model. **(C)** Metabolites located at the extremes of the plot have the greatest contribution to group separation.

The demographic and clinical characteristics of all participants are summarized in Supplementary Table 1. No significant differences were observed among PD subgroups in terms of age and sex distribution, while clinical scale scores reflected expected differences in disease severity and symptom profiles. Metabolite profiling using LC–MS/MS resulted in the identification of 2,601 metabolites across all experimental groups. As shown in Supplementary Figure 12, QC samples clustered tightly in PCA space and showed high pairwise correlations, indicating acceptable analytical stability and reproducibility across the LC–MS/MS runs. To explore global metabolic patterns among PD, PD + RBD, PD + insomnia, and healthy controls (HC), multivariate analyses including PCA, PLS-DA, and OPLS-DA were performed in an exploratory framework. The PCA score plot (Figure 1) illustrated overall variance structure and global metabolic trends among groups. Partial overlap was observed among PD subgroups, while PD and HC samples showed differing distributions, suggesting group-related metabolic variation. PCA results were interpreted as descriptive and exploratory rather than inferential. PLS-DA score plots (Figure 2) further visualized group-associated metabolic patterns, particularly between PD and HC along the first latent component. OPLS-DA analysis (Supplementary Figures 1–3) was applied to enhance interpretability by separating predictive variance related to group differences from orthogonal variance unrelated to classification. Given the high dimensionality of the data and limited sample size, separation observed in supervised models was interpreted cautiously and considered hypothesis-generating.

Volcano plots were generated to visualize differential metabolite trends by integrating fold change and statistical significance (Supplementary Figure 4). Metabolites exhibiting large magnitude changes and low *p*-values were highlighted, facilitating the identification of candidates potentially associated with group-related metabolic variation. Metabolites such as 3-O-Methyl-dopa, ceramides, and glutamic acid appeared at the extremes of the distribution, reflecting pronounced abundance shifts across groups, some of which may be influenced by clinical factors including medication exposure. Forest plots (Supplementary Figure 5) further illustrated log_2_-transformed fold changes for selected metabolites identified through supervised analyses. Based on combined statistical and fold-change thresholds, 50 metabolites showing notable variation were shortlisted for downstream exploratory analyses. These metabolites were mainly involved in amino acid metabolism, lipid-related pathways, and redox-associated processes, suggesting broad metabolic perturbations associated with PD and its clinical subtypes.

### 3.2 Multilevel feature analysis based on metabolite distribution and correlation

**Figure 3:**
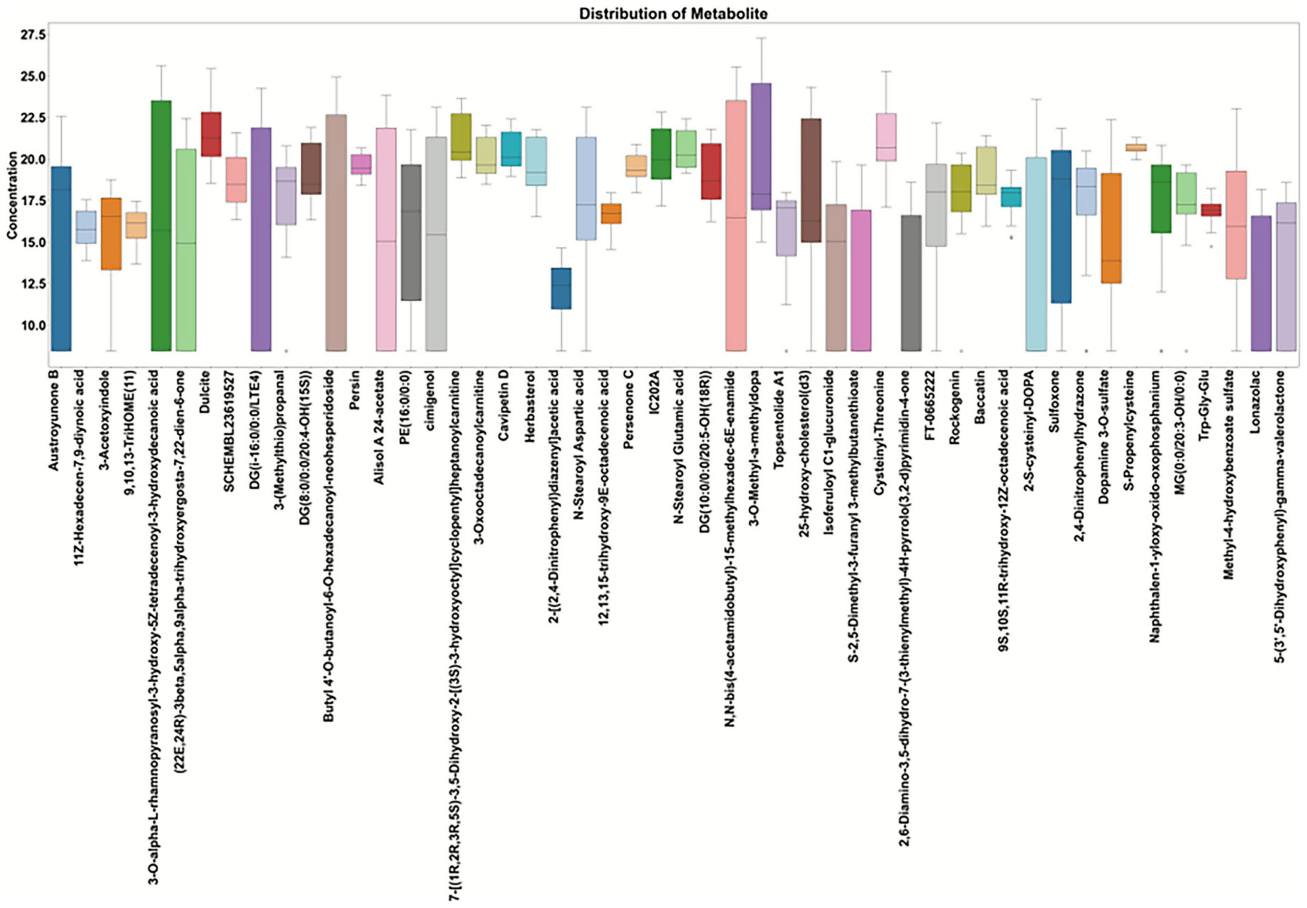
Boxplots of plasma metabolite concentration distributions in HGs and PD. Each boxplot represents the concentration distribution of a specific metabolite, including the median, inter-quartile range, extreme values, and outliers. The horizontal axis denotes the names of the metabolites, while the vertical axis indicates concentration values.

To further investigate the metabolic variations between experimental groups, we employed boxplots and correlation analysis to visualize the distribution of key metabolites and their interrelationships. Boxplots were used to assess the concentration distributions of the most significantly altered metabolites in the plasma of HC and PD (Figure 3). The boxplots revealed that certain metabolites, such as Uric acid, 3-O-Methyl-dopa, and S-2,5-Dimethyl-3-furanyl 3-methylbutanethioate, exhibited distinct concentration ranges between PD and HC. For example, 3-O-Methyl-dopa showed a marked increase in concentration in PD patients. Given that this metabolite is closely related to dopaminergic treatment, its elevation likely reflects combined effects of disease status and medication exposure rather than intrinsic disease pathology alone. Conversely, metabolites like glutamic acid, glycine, and D-mannose were downregulated in PD patients, indicating metabolic disruptions related to amino acid metabolism and energy homeostasis. The variation observed in the boxplots highlighted potential biomarkers for PD that could distinguish the disease from healthy individuals.

**Figure 4:**
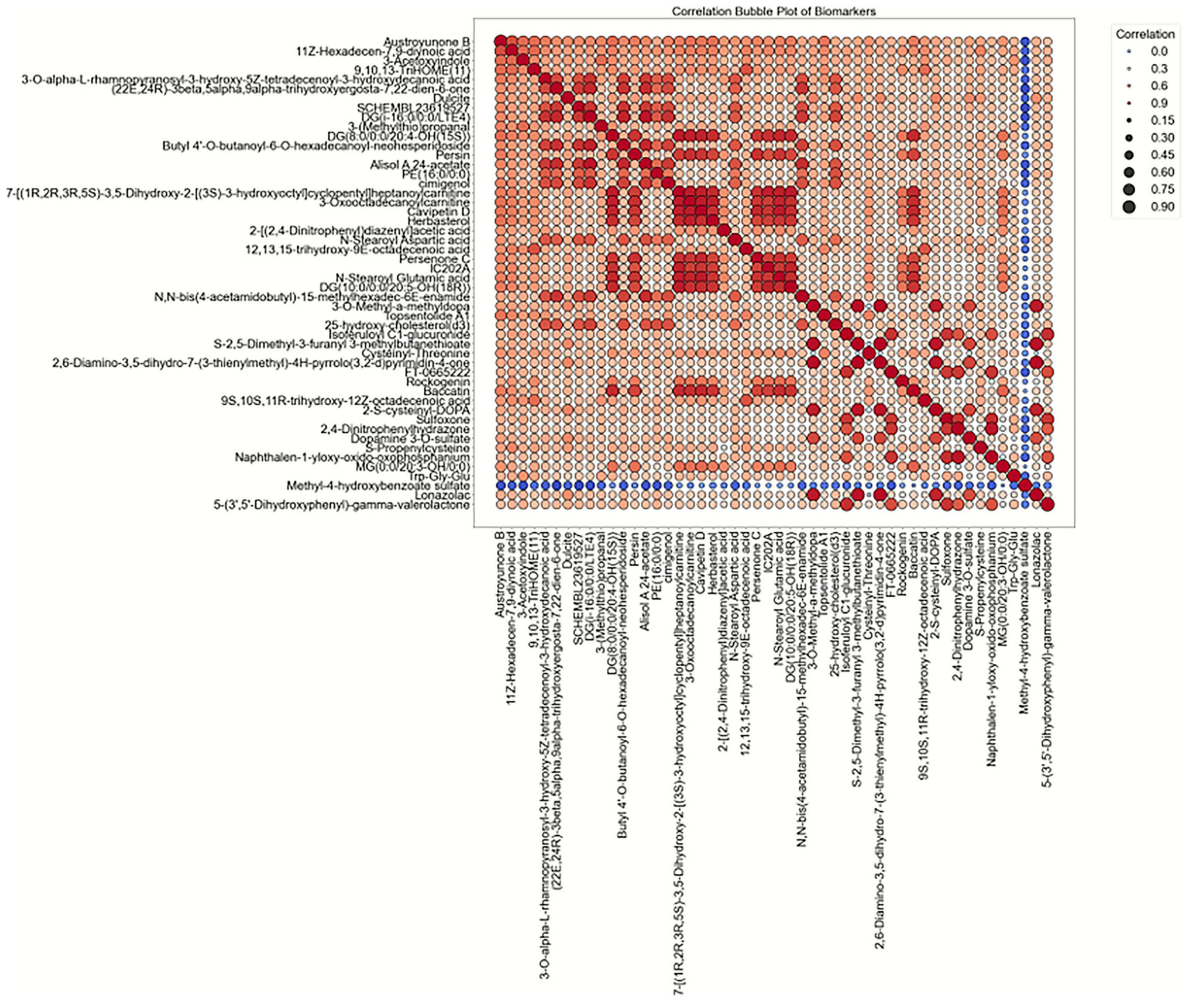
Correlation bubble plot of biomarkers in HC and PD. This plot illustrates the pairwise Pearson correlation coefficients among plasma metabolites. Each circle represents the correlation between a pair of metabolites, with the color indicating the direction (red for positive, blue for negative), and the size representing the magnitude of the correlation. Dark red circles indicate strong positive correlations, while dark blue circles indicate strong negative correlations. The diagonal line reflects self-correlation (value = 1).

In addition to the boxplots, a correlation bubble plot was generated to examine the pairwise relationships between metabolites (Figure 4). This analysis revealed complex co-variation patterns within the metabolic networks. Strong positive correlations were observed between metabolites such as Phenoperidine and Uric acid, suggesting shared metabolic pathways or co-regulation within metabolic networks. For instance, the positive correlation between these metabolites could indicate that they are involved in a common regulatory mechanism or biosynthetic pathway. On the other hand, several negative correlations were identified between metabolites like S-2,5-Dimethyl-3-furanyl 3-methylbutanethioate and lipid-related metabolites, such as phosphatidylcholine and sphingomyelin, suggesting that these metabolites may play opposing roles within cellular metabolic processes. The bubble plot also highlighted metabolites with weak or near-zero correlations, implying that these metabolites operate independently within their respective metabolic pathways. The interrelationships between metabolites, as revealed by the correlation analysis, provided valuable insights into the systemic metabolic changes in PD and its subtypes. These findings emphasize the complexity of metabolic dysregulation in PD and highlight the importance of considering both positive and negative correlations in the context of disease classification. The next step involved using these metabolite distribution patterns to identify key features for machine learning models, which were used for classification and prediction of PD subtypes.

### 3.3 Constructing PD disease prediction models using machine learning

To further explore metabolic patterns associated with Parkinson’s disease and its clinical subtypes, machine-learning analyses were conducted within a proof-of-concept and exploratory framework. Based on the 50 metabolites identified through prior statistical and multivariate analyses, several commonly used supervised learning algorithms, including Random Forest, Support Vector Machine (SVM), and Logistic Regression, were applied to assess internal pattern separation within the dataset. Each algorithm generated a ranked list of features contributing to model separation, with partial overlap observed across methods. Random Forest feature importance analysis (Supplementary Figures 6–8) highlighted metabolites such as 11Z-Hexadecen-7,9-diynoic acid, Austrugynone B, and 3-Acetoxyindole as influential variables. The SVM model emphasized metabolites including (22E,24R)-3β,5*α*,9α-trihydroxyergosta-7,22-dien-6-one and 3-O-Methyl-α-methyldopa, while Logistic Regression assigned higher coefficients to metabolites such as Methyl-4-hydroxybenzoate sulfate and Dulcitol. Notably, several metabolites, including 3-Acetoxyindole and 11Z-Hexadecen-7,9-diynoic acid, were consistently identified across multiple algorithms, suggesting relative stability in feature selection under different modelling assumptions.

**Figure 5:**
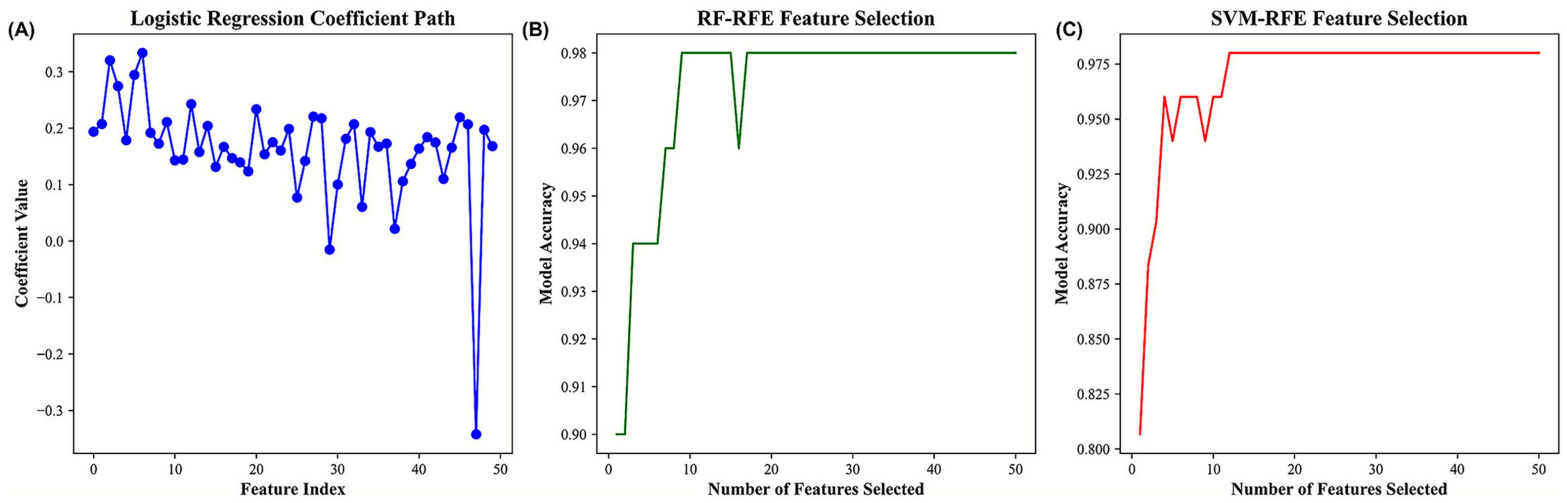
Feature selection across machine learning models. **(A)** Logistic regression coefficient path. **(B)** Random forest recursive feature elimination (RF-RFE). **(C)** Support vector machine recursive feature elimination (SVM-RFE).

**Figure 6:**
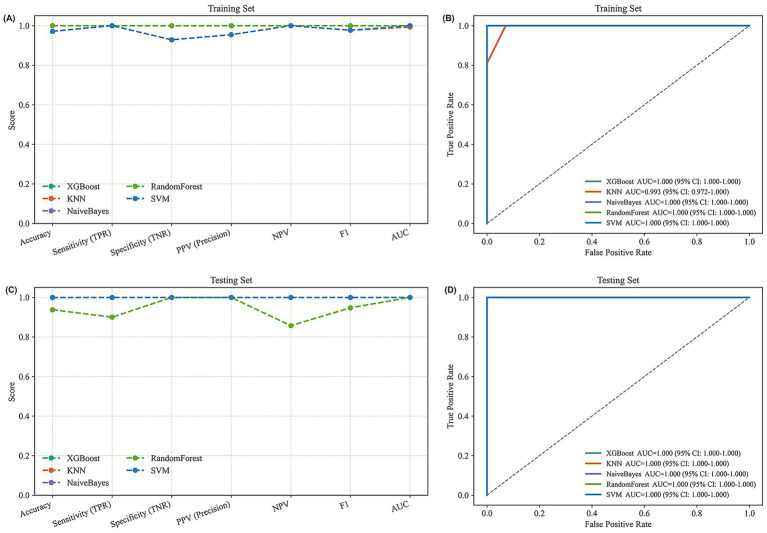
**(A)** Performance metrics of the training set for HC versus PD. **(B)** ROC curve of the training set for HC versus PD. **(C)** Performance metrics of the testing set for HGs versus PD. **(D)** ROC curve of the testing set for HGs versus PD.

To further explore feature stability and reduce dimensionality, recursive feature elimination (RFE) was applied using three different algorithms, including Logistic Regression (LR-RFE), Random Forest (RF-RFE), and Support Vector Machine (SVM-RFE). This procedure systematically reduced the feature set to identify metabolites contributing most consistently to internal model separation. Across all three approaches, model performance remained relatively stable as the number of selected features decreased, suggesting that a limited subset of metabolites captured a substantial proportion of the internal data structure. The coefficient paths derived from Logistic Regression indicated that certain variables consistently contributed to model separation, while the feature selection curves from RF-RFE and SVM-RFE suggested that near-plateau performance could be achieved using relatively small feature sets. These observations indicate that feature reduction may help mitigate noise in high-dimensional metabolomics data; however, improvements in generalizability cannot be inferred without external validation. By intersecting the outputs of the three RFE-based approaches, a core set of metabolites—including 3-Acetoxyindole, 9,10,13-TriHOME (11), and Dulcitol—was identified (Figure 5). These metabolites were repeatedly selected across Logistic Regression, Random Forest, and SVM models, suggesting relative stability in feature selection under different modeling assumptions. Rather than serving as definitive biomarkers, these metabolites are considered candidate features for further validation. To evaluate internal model performance, multiple metrics—including accuracy, sensitivity, specificity, F1 score, and area under the receiver operating characteristic curve (AUC)—were calculated under internal validation using training and testing partitions. Several machine-learning models, including XGBoost, Random Forest, SVM, K-Nearest Neighbors, and Naive Bayes, exhibited strong internal separation, with high performance metrics observed in both partitions (Figure 6; Tables 1, 2). However, given the limited sample size and absence of an independent external cohort, these performance estimates should be interpreted as optimistic and exploratory rather than indicative of generalizable predictive accuracy.

**Table 1:** Performance metrics for five machine learning models on the training set.

Model	Accuracy	TPR	TNR	PPV	NPV	F1	AUC
XGBoost	1	1	1	1	1	1	1
KNN	0.9714	1	0.9286	0.9545	1	0.9767	0.9932
NaiveBayes	0.9714	1	0.9286	0.9545	1	0.9767	1
RandomForest	1	1	1	1	1	1	1
SVM	0.9714	1	0.9286	0.9545	1	0.9767	1
XGBoost	1	1	1	1	1	1	1

**Table 2:** Performance metrics for five machine learning models on the testing set.

Model	Accuracy	TPR	TNR	PPV	NPV	F1	AUC
XGBoost	1	1	1	1	1	1	1
KNN	1	1	1	1	1	1	1
NaiveBayes	1	1	1	1	1	1	1
RandomForest	0.9375	0.9	1	1	0.8571	0.9474	1
SVM	1	1	1	1	1	1	1
XGBoost	1	1	1	1	1	1	1

**Figure 7:**
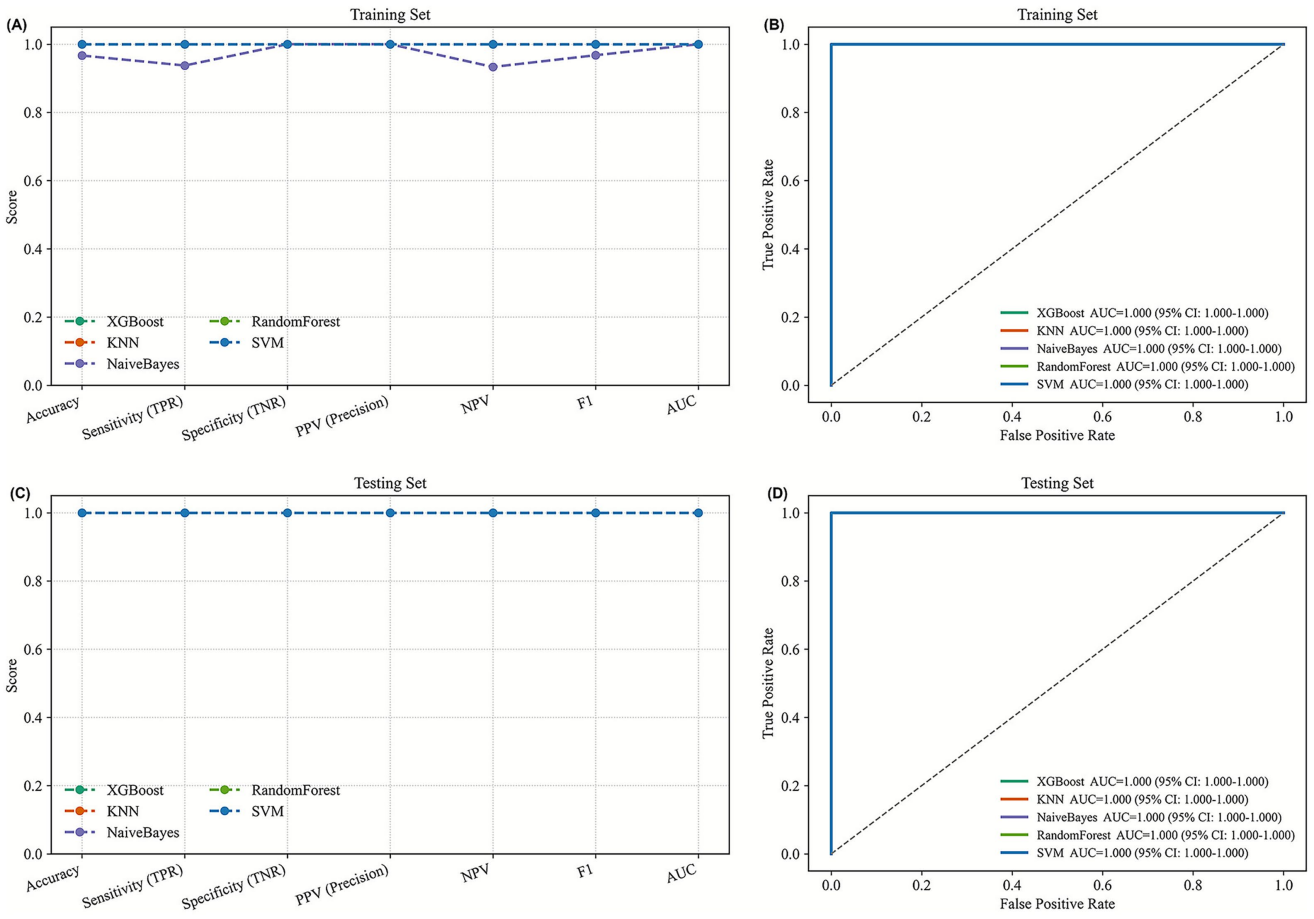
**(A)** Performance metrics of the training set for HC versus PD + insomnia classification. **(B)** ROC curve of the training set for HC versus PD + insomnia. **(C)** Performance metrics of the testing set for HC versus PD + insomnia. **(D)** ROC curve of the testing set for HC versus PD + insomnia.

Using the RFE approach, 16 metabolites were further identified to explore metabolic patterns differentiating PD + insomnia from HC. These metabolites included Alternariol, Fentiazac, Biurea, Piroximone, 3-O-alpha-L-rhamnopyranosyl-3-hydroxy-5Z-tetradecenoyl-3-hydroxydecanoic acid, Algestone, ONO-2235, O-Mustard, SCHEMBL1706204L-Methionine, NOTA, S-Allyl-L-cysteine, 5-Ethyl-2-hexyl-4-methyloxazole, 5-Methyl-3E-hexen-2-one, 3-Sulfodeoxycholic acid, 3-[4-(sulfooxy)phenyl]propanoic acid, Tyrosine, and 3-hydroxy-O-methyl-. supervised models constructed using these metabolites demonstrated high internal performance in the training set. Several models, including XGBoost, Random Forest, and SVM, achieved high AUC values in the testing partition, while KNN and Naive Bayes showed slightly lower but still consistent performance (Figure 7). These findings suggest that the selected metabolic features capture group-related patterns associated with PD and PD-related insomnia. However, given the exploratory nature of the analysis and the absence of external validation, these results should be interpreted cautiously and warrant further confirmation in larger, independent cohorts.

### 3.4 Metabolic profiling of Parkinson’s disease with distinct non-motor manifestations

Building upon the observed metabolic differences between healthy controls (HC) and patients with Parkinson’s disease (PD), we further explored metabolic variation among PD patients presenting with different non-motor symptoms. Specifically, metabolic patterns associated with rapid eye movement sleep behavior disorder (PD + RBD) and insomnia (PD + insomnia) were examined to investigate symptom-related metabolic alterations. This analysis reflects the clinical heterogeneity of PD and aims to explore metabolomic features potentially associated with distinct symptom presentations.

To further examine metabolic patterns related to RBD, machine-learning models were applied to explore differences between PD patients with and without this comorbidity. Using recursive feature elimination (RFE), 16 metabolites were identified that consistently contributed to internal model separation, including O-Mustard, SCHEMBL1706204L-Methionine, NOTA, S-Allyl-L-cysteine, 5-Ethyl-2-hexyl-4-methyloxazole, 5-Methyl-3E-hexen-2-one, 3-Sulfodeoxycholic acid, 3-[4-(sulfooxy)phenyl]propanoic acid, Tyrosine, 3-hydroxy-O-methyl-, 3-Acetylphenol sulfate, (22E)-1α,3β-Dihydroxychola-5,16,22-trien-24-oic acid, Prehumulinic acid, Amotosalen, 5β-Chola-8(14),11-dien-24-oic acid, and S-2,5-Dimethyl-3-furanyl 3-methylbutanethioate. These metabolites were used as candidate features for supervised modeling to explore internal separation between PD patients with and without RBD. As shown by the ROC curves and performance metrics from the training and testing partitions (Supplementary Figure 9), several models, including Random Forest, XGBoost, and Support Vector Machine (SVM), exhibited high internal performance. Although near-perfect AUC values were observed for some models, these results were interpreted cautiously due to the limited sample size and internal validation design. K-Nearest Neighbors and Naive Bayes models demonstrated slightly lower AUC values but showed generally consistent performance across evaluation metrics.

To explore metabolic patterns associated with insomnia in PD, machine-learning analyses were further conducted using the 50 metabolites identified through feature selection. Several metabolites, including Alternariol, Fentiazac, Biurea, Piroximone, 3-O-alpha-L-rhamnopyranosyl-3-hydroxy-5Z-tetradecenoyl-3-hydroxydecanoic acid, Algestone, and ONO-2235, were identified as contributing to internal separation between PD patients with and without insomnia. Supervised models demonstrated relatively consistent internal performance across multiple algorithms (Supplementary Figure 10), suggesting that these metabolic features may be associated with insomnia-related manifestations in PD. Overall, these findings provide an exploratory basis for future validation studies in larger, independent cohorts.

### 3.5 Symptom-associated metabolite profiles in PD

**Figure 8:**
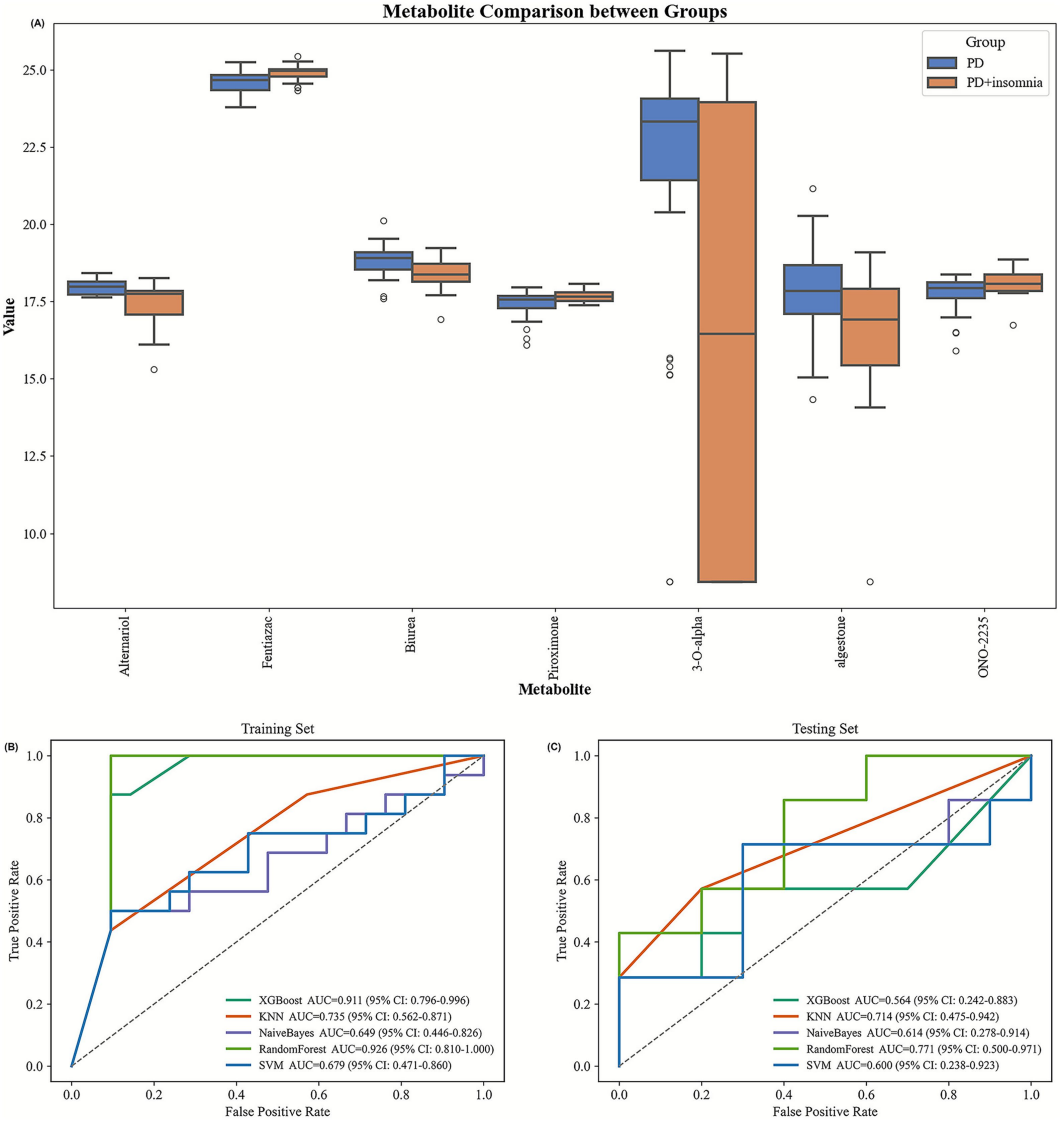
**(A)** Boxplots illustrate the distribution of metabolite concentrations between PD and PD + insomnia groups. **(B)** ROC curves of 3-O-alpha-L-rhamnopyranosyl-3-hydroxy-5Z-tetradecenoyl-3-hydroxydecanoic acid in the training set. **(C)** ROC curves of 3-O-alpha-L-rhamnopyranosyl-3-hydroxy-5Z-tetradecenoyl-3-hydroxydecanoic acid in the testing set.

In addition to statistical analyses, boxplots (Figure 8) were generated to illustrate the concentration distributions of representative metabolites between PD patients with and without insomnia. The results showed observable differences in metabolite abundance, particularly for 3-O-alpha-L-rhamnopyranosyl-3-hydroxy-5Z-tetradecenoyl-3-hydroxydecanoic acid, which exhibited higher levels in PD patients presenting with insomnia. This metabolite therefore emerged as a candidate feature potentially associated with sleep disturbances in PD, based on its concentration differences between the two groups. ROC analyses were further conducted to explore the ability of this metabolite to contribute to group separation. In the training dataset, Random Forest and XGBoost models achieved AUC values of 0.911 and 0.925, respectively. A modest decrease in performance was observed in the testing dataset, where KNN yielded the highest AUC value (0.714), suggesting limited generalizability and potential overfitting. Nevertheless, these results indicate that 3-O-alpha-L-rhamnopyranosyl-3-hydroxy-5Z-tetradecenoyl-3-hydroxydecanoic acid may be associated with insomnia-related metabolic variation in PD and warrants further investigation in larger cohorts.

Similarly, S-2,5-Dimethyl-3-furanyl 3-methylbutanethioate was identified as a metabolite differing between PD patients and those with rapid eye movement sleep behavior disorder (PD + RBD) (Supplementary Figure 11). This metabolite showed elevated levels in PD + RBD patients, suggesting potential alterations in metabolic pathways associated with this comorbidity. Supervised models, including Random Forest and SVM, demonstrated high performance in the training dataset and moderate yet consistent performance in the testing dataset, indicating internal separation but limited external inference. Collectively, these results highlight metabolic heterogeneity among PD patients with distinct non-motor symptoms. The identification of metabolites associated with comorbid insomnia and RBD provides exploratory insights into symptom-related metabolic variation in PD and offers a basis for future validation studies, rather than definitive diagnostic or therapeutic conclusions.

## 4 Discussion

### 4.1 Resource identification initiative

PD is a progressive neurodegenerative disorder that remains challenging to diagnose accurately, particularly in its early stages. Variability in motor symptoms and the reliance on subjective clinical assessments often contribute to diagnostic delays. In this study, we applied a machine learning–assisted metabolomics approach to explore metabolic alterations associated with PD and its non-motor manifestations. By integrating high-resolution LC–MS/MS with multivariate statistical analyses and supervised modeling, we investigated metabolic features that may help characterize PD and symptom-related heterogeneity within the studied cohort.

Our findings support the presence of widespread metabolic alterations associated with PD. Comprehensive LC–MS/MS profiling identified 2,601 metabolites, and exploratory multivariate analyses, including PCA, PLS-DA, and OPLS-DA, revealed group-related metabolic patterns between PD patients and healthy controls. In addition, differences in metabolic profiles were observed among PD patients with comorbid insomnia and RBD. These observations are consistent with previous studies suggesting that PD-related metabolic disturbances extend beyond dopaminergic dysfunction and may involve broader alterations in lipid metabolism, amino acid turnover, oxidative stress responses, and energy homeostasis. Several metabolites showed symptom-associated variation, providing exploratory insights into the metabolic basis of non-motor manifestations in PD.

Machine learning approaches were incorporated to assist feature selection and pattern recognition in this high-dimensional dataset. By applying RFE in combination with Logistic Regression, Random Forest, and Support Vector Machine models, we identified subsets of metabolites that consistently contributed to internal group separation. Rather than establishing definitive classifiers, these analyses were intended to explore structured metabolic patterns and highlight candidate features associated with PD and its symptom subtypes. The results demonstrate the utility of machine learning as an exploratory tool for identifying biologically relevant signals within complex metabolomic data.

An additional observation of this study is the identification of metabolites potentially associated with specific non-motor symptoms in PD. For example, 3-O-alpha-L-rhamnopyranosyl-3-hydroxy-5Z-tetradecenoyl-3-hydroxydecanoic acid and S-2,5-Dimethyl-3-furanyl 3-methylbutanethioate exhibited differential abundance patterns between PD patients with insomnia and those with RBD. These findings suggest that distinct metabolic alterations may underlie different sleep-related manifestations in PD. Given the increasing recognition of non-motor symptoms as major contributors to disease burden and reduced quality of life, such symptom-associated metabolic features warrant further investigation in larger and independent cohorts.

The performance of machine learning models was evaluated using multiple metrics, including accuracy, sensitivity, specificity, and area under the ROC curve (AUC). Several models demonstrated high internal performance within the training and testing partitions. However, given the limited sample size, high feature-to-sample ratio, and lack of external validation, these results should be interpreted as exploratory and reflective of internal data structure rather than generalizable predictive accuracy. Future studies incorporating larger, well-characterized cohorts and independent validation will be essential to determine the clinical relevance and translational potential of the identified metabolic features.

## 5 Conclusion

This study provides evidence supporting the potential value of integrating metabolomics and machine learning to explore metabolic characteristics associated with Parkinson’s disease. By examining metabolic profiles in PD patients with and without comorbid conditions such as insomnia and RBD, we identified symptom-associated metabolic patterns related to these non-motor manifestations. The application of advanced algorithms, particularly RFE, facilitated the identification of metabolite features that consistently contributed to internal pattern separation across models. Our approach contributes to the understanding of metabolic disturbances in PD and highlights the utility of data-driven methods for characterizing symptom-related metabolic variation. Rather than establishing definitive diagnostic tools, the identified metabolic features should be regarded as candidate markers that may reflect underlying biological differences among PD subgroups. The observed model performance reflects internal separability within the analyzed dataset and should be interpreted in light of the exploratory study design and limited sample size. Overall, this work underscores the importance of considering metabolic heterogeneity and non-motor symptoms in PD research, particularly in relation to individualized disease characterization. Future studies should aim to validate these candidate metabolic features in larger and more diverse populations and to further refine modeling approaches for longitudinal investigation. With appropriate external validation, integrative metabolomics and machine learning strategies may contribute to improved understanding of symptom progression and support the development of more tailored approaches to PD management.

## Data Availability

The datasets presented in this study can be found in online repositories. The names of the repository/repositories and accession number(s) can be found in the article/Supplementary material.
