# Supplementary material for: Machine learning-guided analysis of metabolomic alterations in Parkinson’s disease with comorbid symptoms
Source: Front Aging Neurosci. 2026 Jan 13;17:1753016. doi: 10.3389/fnagi.2025.1753016 (PMC12835381; doi:10.3389/fnagi.2025.1753016)
Supplement: Supplementary file 1 [file Data_Sheet_1.docx]

Supplementary Material

# Supplementary Figures


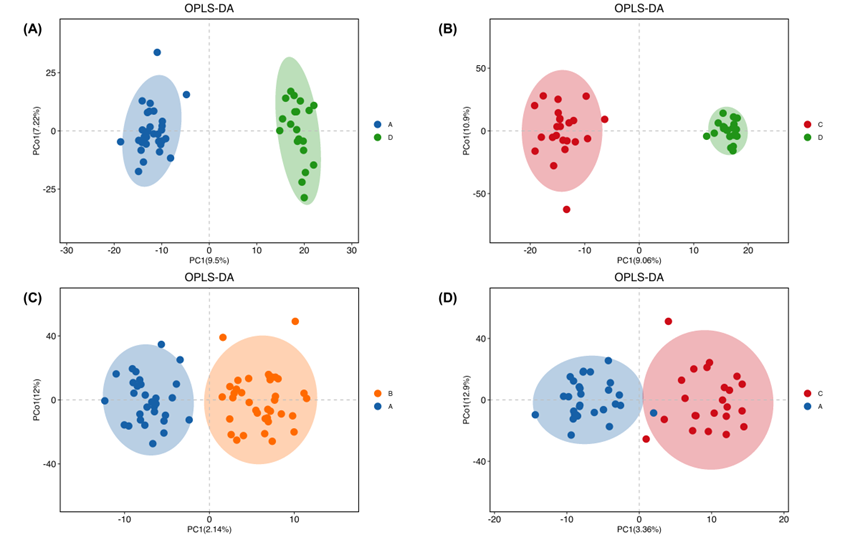


**Supplementary Figure 1.** OPLS-DA score plot. (A) OPLS-DA score plot for PD and HGs. (B) OPLS-DA score plot for PD and PD+insomnia. (C) OPLS-DA score plot for PD and PD+RBD. (D) OPLS-DA score plot for PD and PD+insomnia.


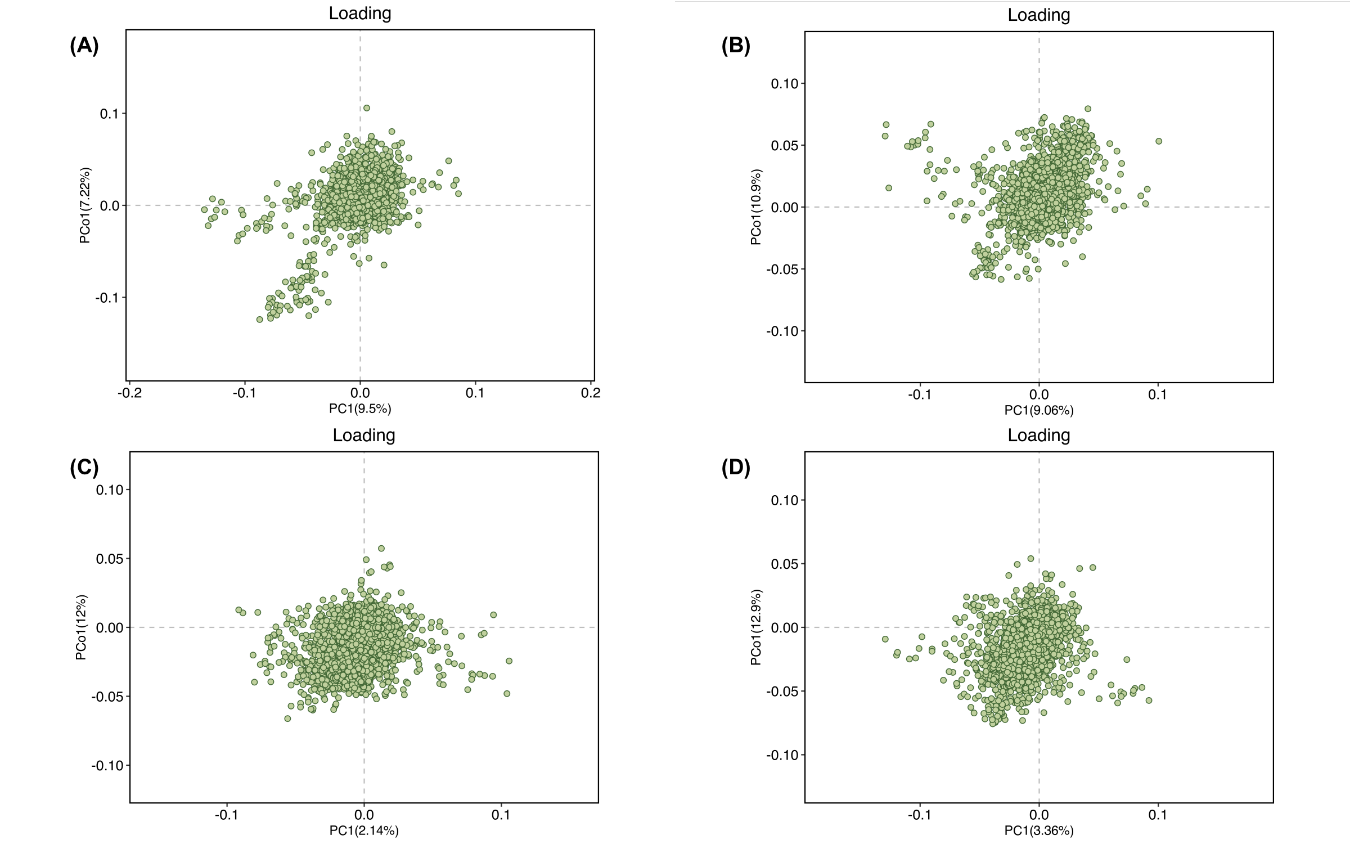


**Supplementary Figure 2.** OPLS-DA Loading Plot. (A) OPLS-DA loading plot for the separation between PD and HGs. (B) OPLS-DA loading plot for the separation between PD and PD+insomnia. (C) OPLS-DA loading plot for the separation between PD and PD+RBD. (D) OPLS-DA loading plot for the sep-aration between PD and PD+insomnia.


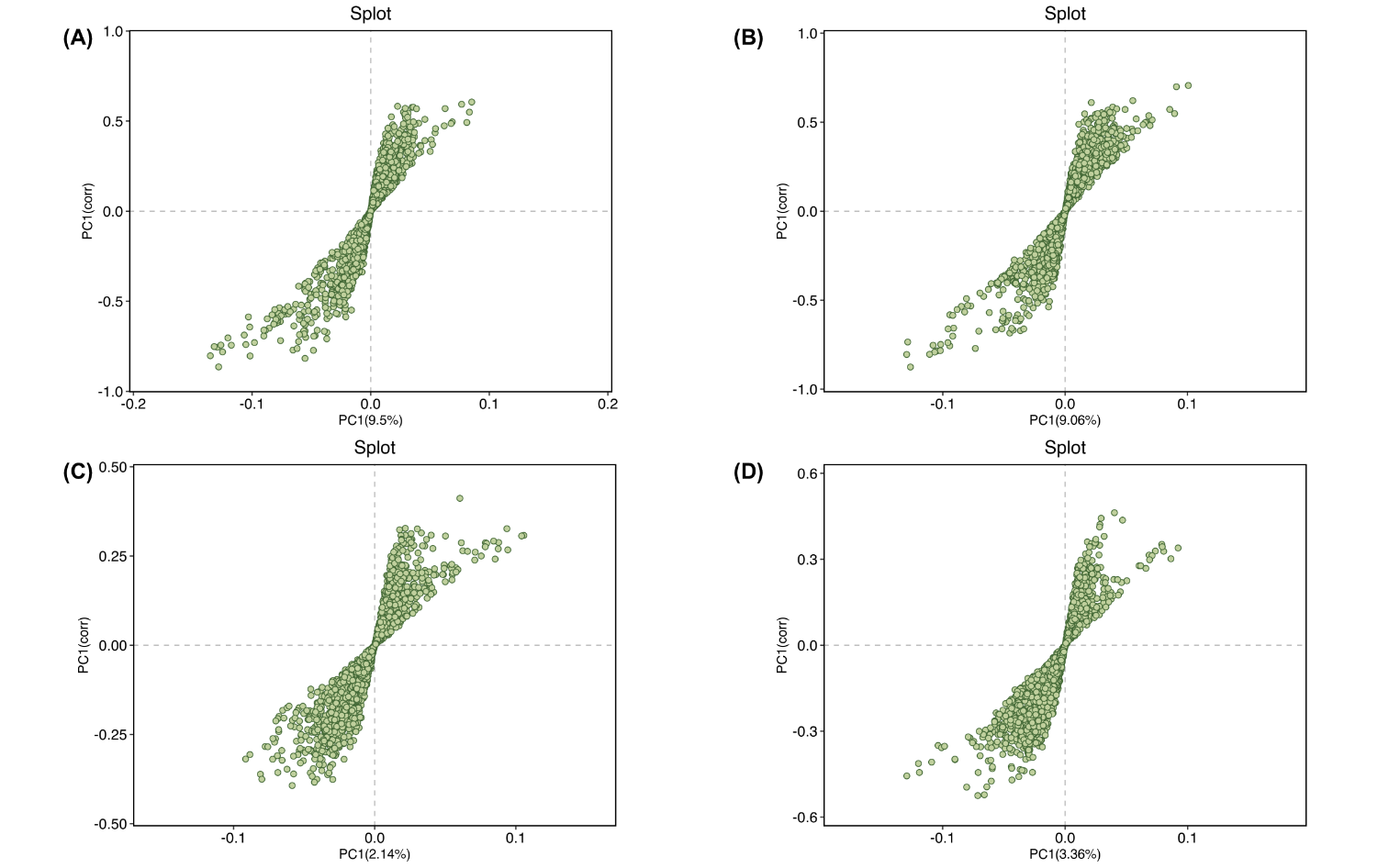


**Supplementary Figure 3.** OPLS-DA S-Plot. (A) OPLS-DA S-plot for the separation between PD and HG. (B) OPLS-DA S-plot for the separation between PD and PD+insomnia. (C) OPLS-DA S-plot for the separation between PD and PD+RBD. (D) OPLS-DA S-plot for the separation between PD and PD+insomnia.


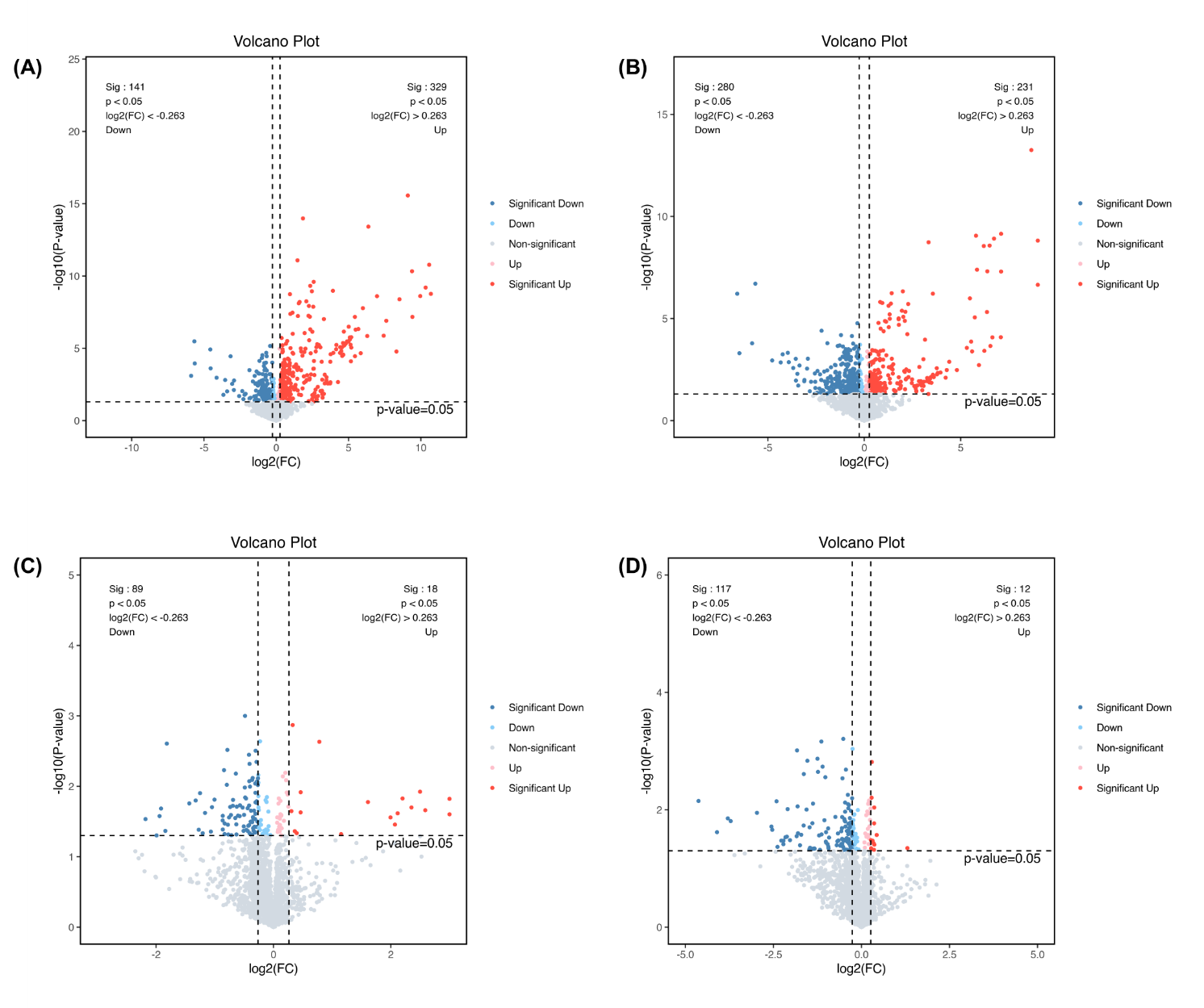


**Supplementary Figure 4.** Volcano Plots for Differential Metabolite Analysis. (A) Volcano plot comparing PD and HG. (B) Volcano plot comparing PD and PD+insomnia. (C) Volcano plot comparing PD and PD+RBD. (D) Volcano plot comparing PD and PD+insomnia.


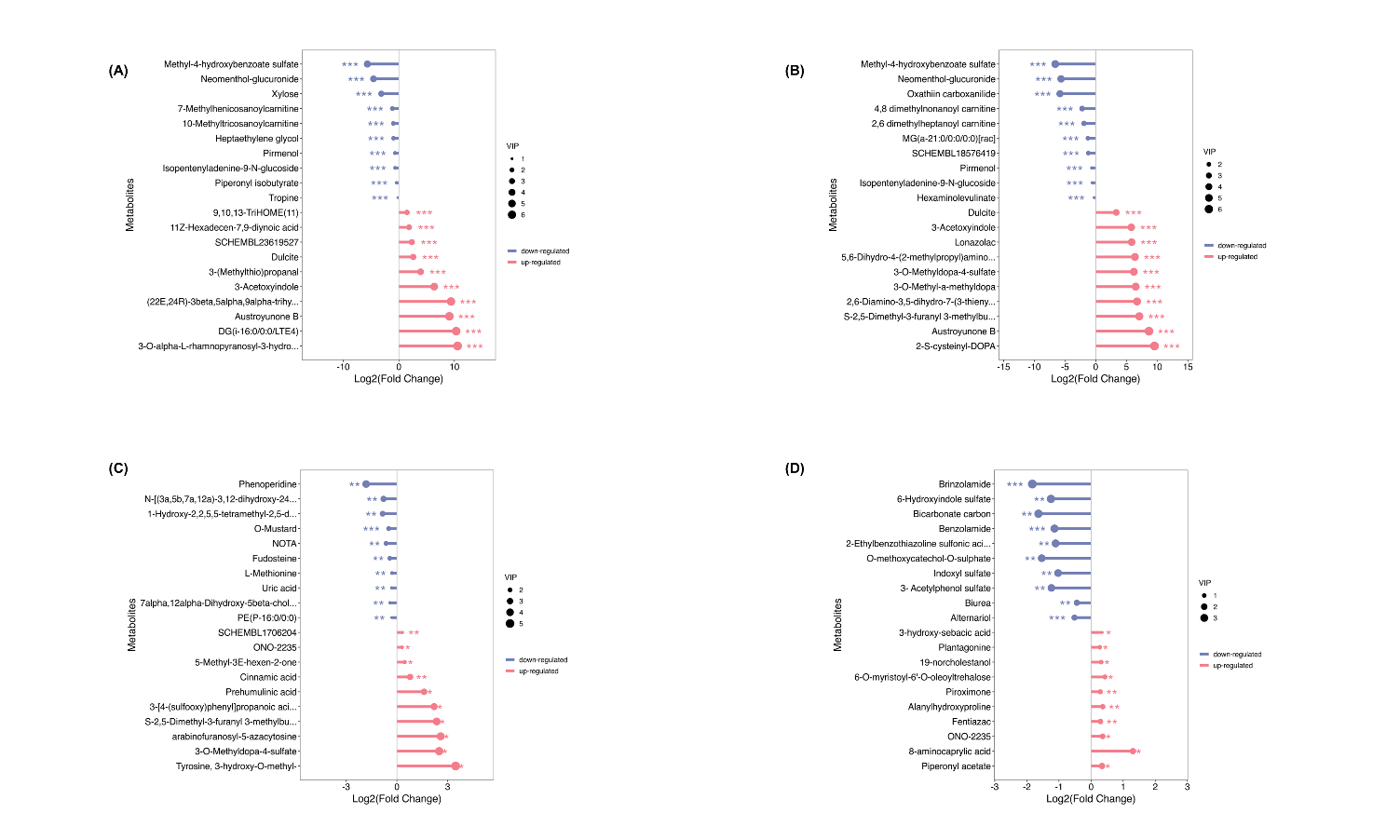


**Supplementary Figure 5.** Forest Plots for Differential Metabolite Analysis. (A) Forest plot showing the log2(fold change) of metabolites in the comparison between PD and HGs. (B) Forest plot comparing PD and PD+insomnia. (C) Forest plot comparing PD and PD+RBD. (D) Forest plot comparing PD and PD+insomnia. The VIP values are indicated by the size of the dots.


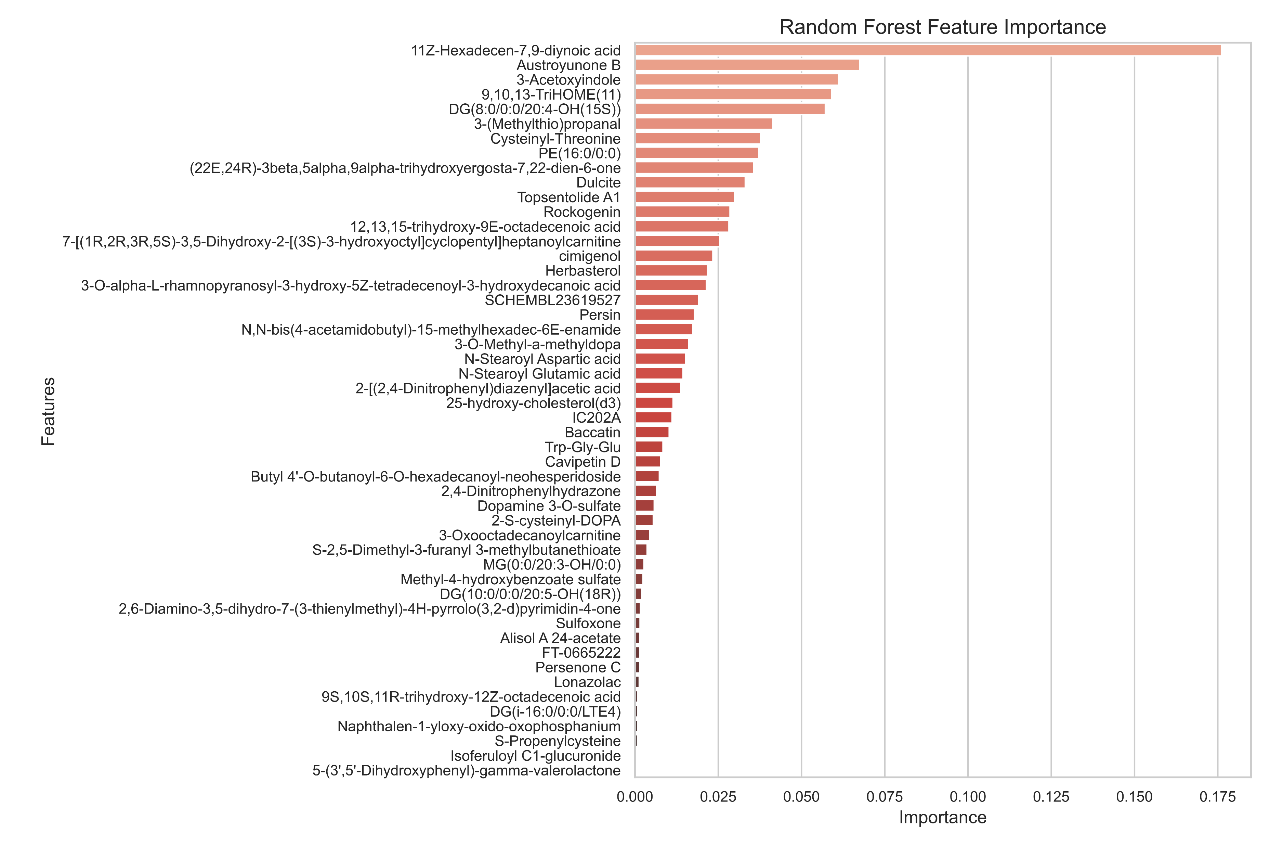


**Supplementary Figure 6.** Feature importance ranking in the Random Forest model


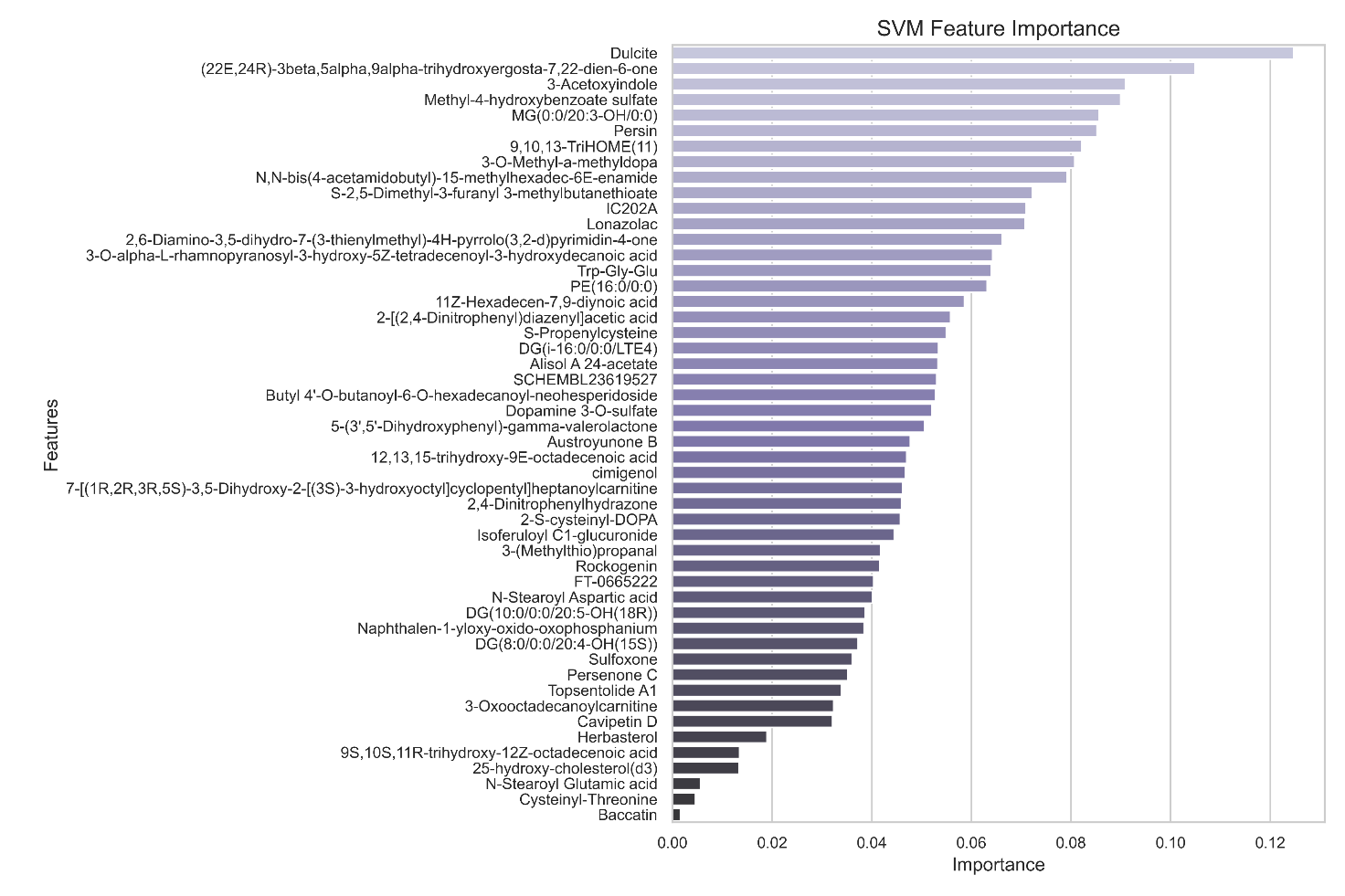


**Supplementary Figure 7.** Feature importance ranking in the Support Vector Machine model.


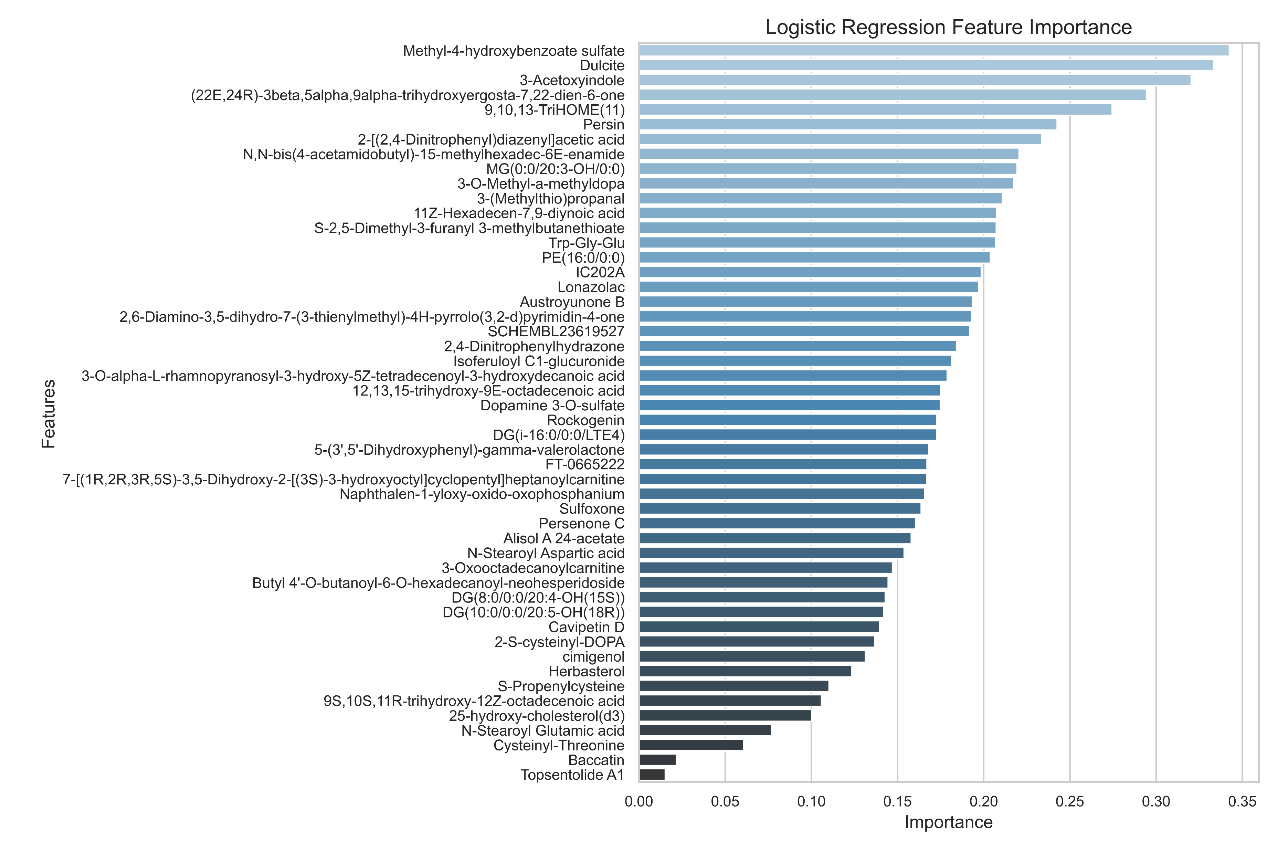


**Supplementary Figure 8.** Feature importance ranking in the Logistic Regression model.


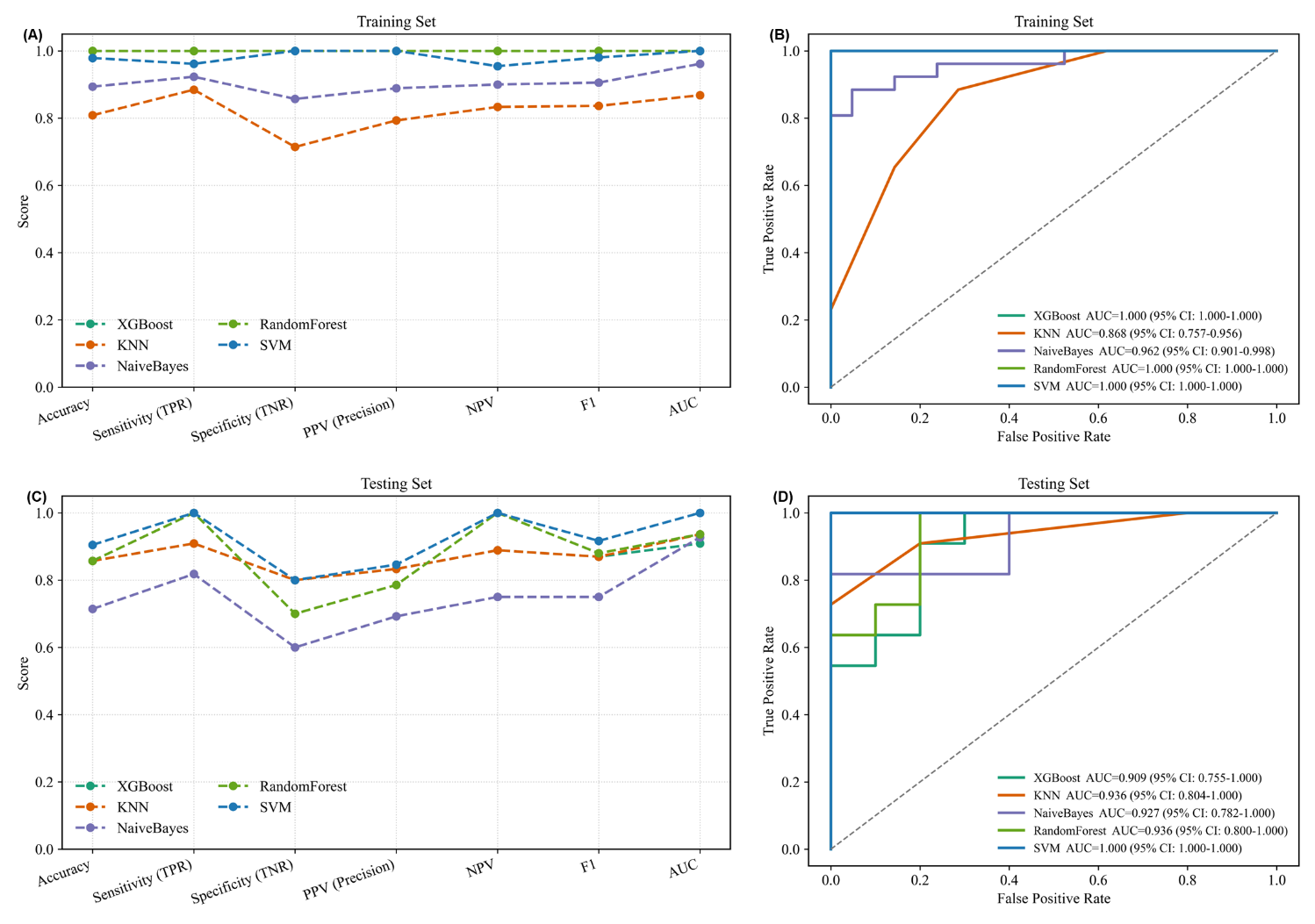


**Supplementary Figure 9.** (A) Performance metrics of the training set for PD versus PD+RBD. (B) ROC curve of the training set for PD versus PD+RBD. (C) Performance metrics of the testing set for PD versus PD+RBD. (D) ROC curve of the testing set for PD versus PD+RBD.


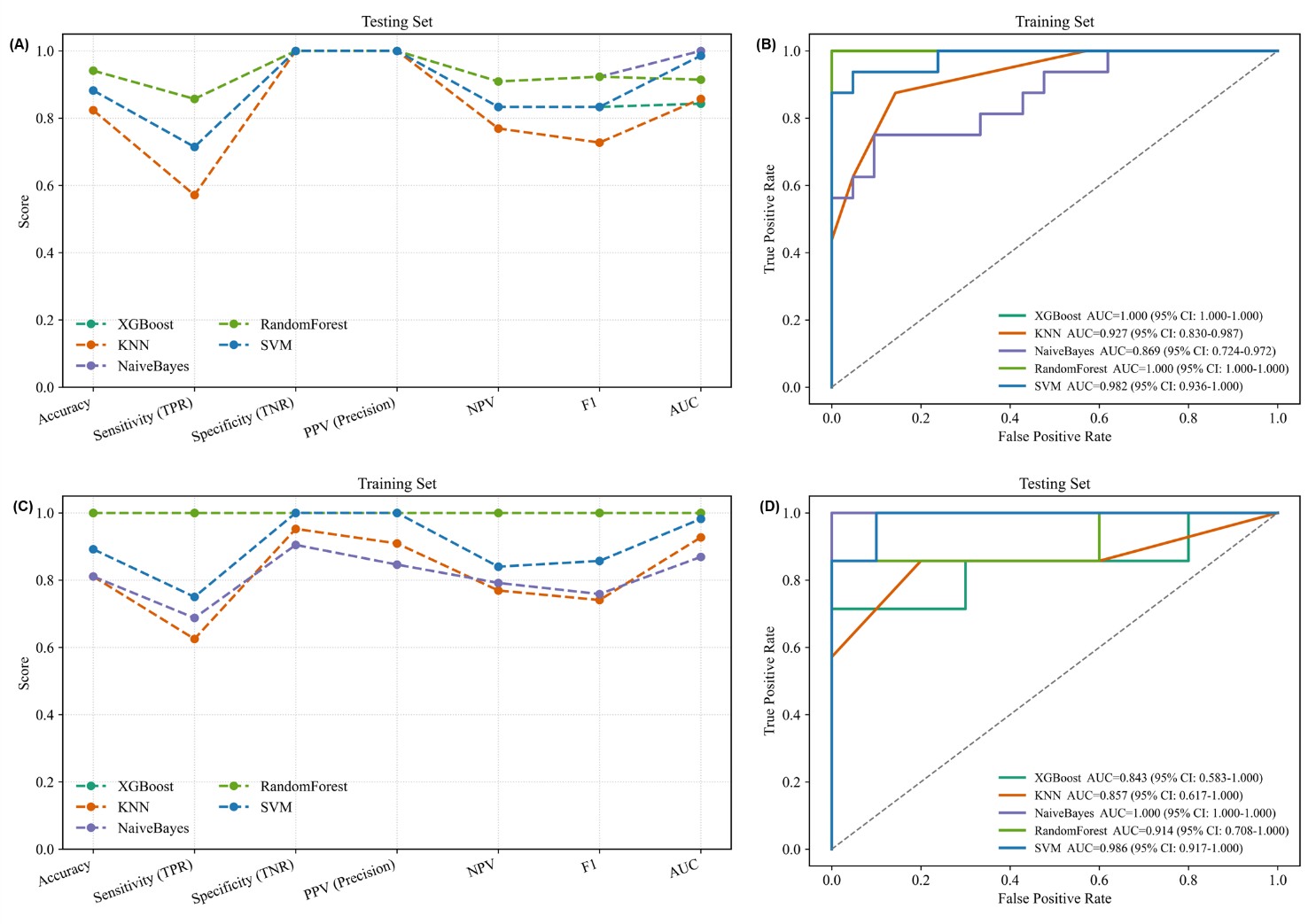


**Supplementary Figure 10.** (A) Performance metrics of the training set for PD versus PD + insomnia. (B) ROC curve of the training set for PD versus PD + insomnia. (C) Performance metrics of the testing set for PD versus PD + insomnia. (D) ROC curve of the testing set for PD versus PD + insomnia.


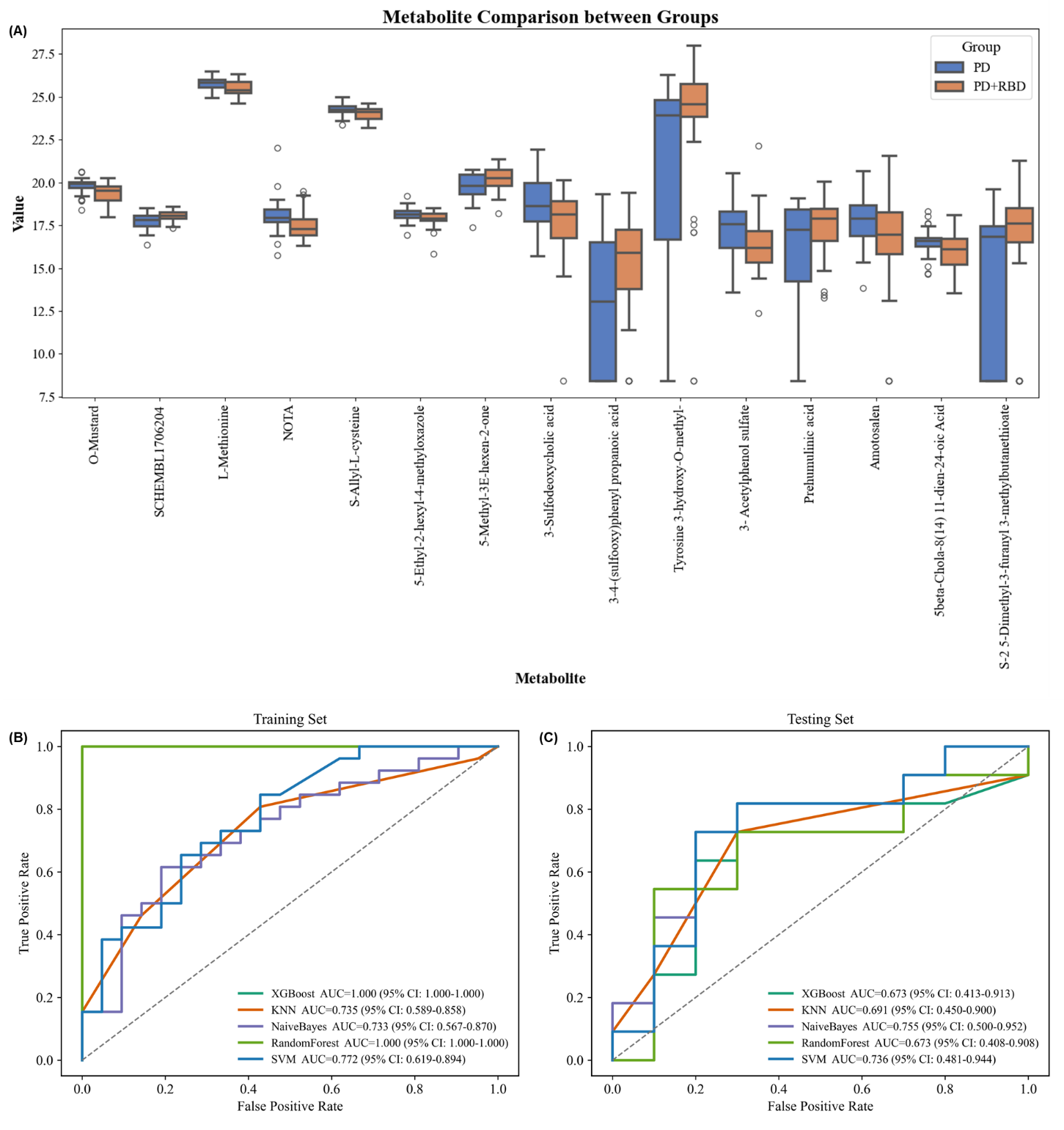


**Supplementary Figure 11.**  (A) Boxplots illustrate the distribution of metabolite concentrations between PD and PD+RBD. (B) ROC curves of S-2,5-Dimethyl-3-furanyl 3-methylbutanethioate, Tyrosine, 3-hydroxy-O-methyl-, and 3-4-(sulfooxy)phenyl propanoic acid in the training set. (C) ROC curves of S-2,5-Dimethyl-3-furanyl 3-methylbutanethioate, Tyrosine, 3-hydroxy-O-methyl-, and 3-4-(sulfooxy)phenyl propanoic acid in the testing set.

**
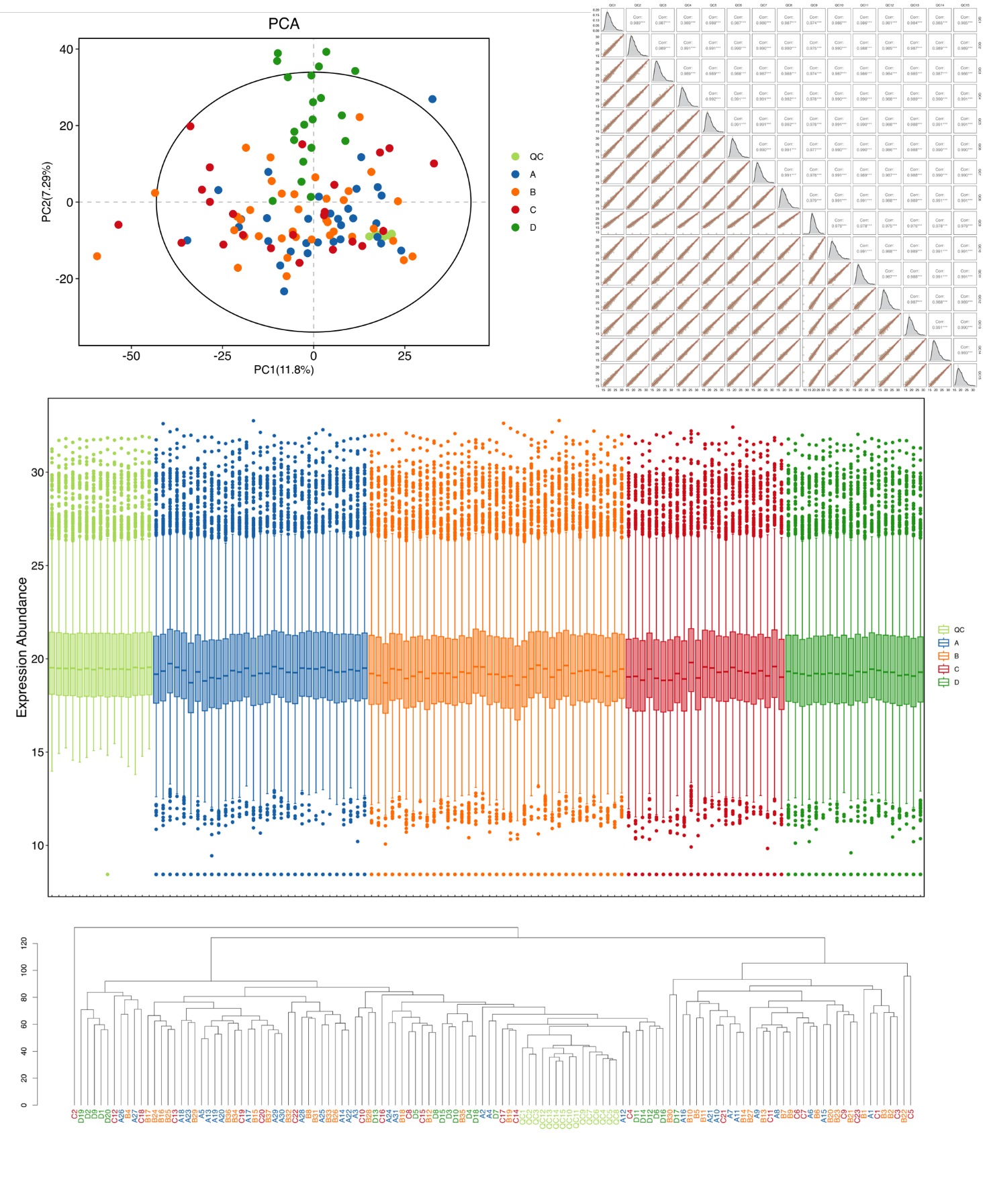
**

**Supplementary Figure 12.** Quality control and batch-effect assessment of metabolomics data. A, Parkinson’s disease (PD); B, Parkinson’s disease with rapid eye movement sleep behavior disorder (PD+RBD); C, Parkinson’s disease with insomnia (PD+insomnia); and D, healthy control (HC).

# Supplementary Tables

**Supplementary Table 1.** Demographic and clinical information of PD patients and healthy controls. PD patients were classified as PD, PD+insomnia, and PD+RBD. H–Y stage, UPDRS score, and dopamine equivalent dose (LEDD) were recorded for PD patients, while these variables were not applicable for healthy controls.

| Samples | Sleep disorder | Gender | Age | Course of the disease(months) | H-Y instalments | UPDRS score | Dopamine equivalent |
| --- | --- | --- | --- | --- | --- | --- | --- |
| Sample 1 | PD | Female | 71 | 0.5 | 2.5 | 41 | 187.5 |
| Sample 2 | PD | Male | 52 | 2 | 2 | 42 | 412.5 |
| Sample 3 | PD | Male | 71 | 1 | 2.5 | 53 | 450 |
| Sample 4 | PD | Male | 60 | 8 | 2.5 | 20 | 312.5 |
| Sample 5 | PD | Male | 50 | 8 | 4 | 78 | 862.5 |
| Sample 6 | PD | Male | 81 | 10 | 4 | 55 | 625 |
| Sample 7 | PD | Male | 83 | 3 | 3 | 50 | 387.5 |
| Sample 8 | PD | Male | 76 | 2 | 2 | 30 | 337.5 |
| Sample 9 | PD | Male | 71 | 4 | 2 | 21 | 187.5 |
| Sample 10 | PD | Female | 82 | 0.5 | 2 | 22 | 337.5 |
| Sample 11 | PD | Female | 75 | 3 | 1 | 30 | 337.5 |
| Sample 12 | PD | Male | 69 | 1 | 2 | 22 | 500 |
| Sample 13 | PD | Male | 48 | 2 | 2 | 46 | 425 |
| Sample 14 | PD | Male | 78 | 2 | 2 | 11 | 187.5 |
| Sample 15 | PD | Male | 82 | 0.5 | 2 | 39 | 187.5 |
| Sample 16 | PD | Male | 70 | 1 | 2.5 | 18 | 50 |
| Sample 17 | PD | Female | 81 | 3 | 2 | 25 | 187.5 |
| Sample 18 | PD | Male | 65 | 10 | 4 | 44 | 575 |
| Sample 19 | PD | Male | 52 | 1 | 1 | 27 | 400 |
| Sample 20 | PD | Male | 69 | 4 | 2 | 21 | 425 |
| Sample 21 | PD | Male | 79 | 2 | 2 | 18 | 425 |
| Sample 22 | PD | Female | 79 | 0.5 | 1 | 21 | 337.5 |
| Sample 23 | PD | Female | 73 | 0.5 | 2 | 13 | 150 |
| Sample 24 | PD | Female | 82 | 1 | 1 | 18 | 187.5 |
| Sample 25 | PD | Male | 69 | 1 | 1 | 16 | 187.5 |
| Sample 26 | PD | Female | 50 | 2 | 1 | 9 | 337.5 |
| Sample 27 | PD | Female | 72 | 1 | 2 | 29 | 450 |
| Sample 28 | PD | Male | 73 | 10 | 3 | 35 | 500 |
| Sample 29 | PD | Female | 71 | 3 | 2.5 | 28 | 500 |
| Sample 30 | PD | Female | 72 | 2 | 3 | 30 | 275 |
| Sample 31 | PD | Female | 71 | 4 | 2.5 | 26 | 187.5 |
| Sample 32 | PD and insomnia | Female | 72 | 30 | 4 | 74 | 187.5 |
| Sample 33 | PD and insomnia | Male | 50 | 1 | 3 | 24 | 462.5 |
| Sample 34 | PD and insomnia | Female | 59 | 4 | 2 | 39 | 350 |
| Sample 35 | PD and insomnia | Male | 68 | 9 | 2 | 28 | 412.5 |
| Sample 36 | PD and insomnia | Female | 56 | 1 | 2.5 | 62 | 412.5 |
| Sample 37 | PD and insomnia | Female | 72 | 6 | 3 | 77 | 650 |
| Sample 38 | PD and insomnia | Male | 66 | 4 | 2 | 31 | 575 |
| Sample 39 | PD and insomnia | Male | 65 | 0.5 | 2 | 16 | 187.5 |
| Sample 40 | PD and insomnia | Male | 72 | 1 | 2.5 | 34 | 337.5 |
| Sample 41 | PD and insomnia | Male | 45 | 2 | 2 | 19 | 237.5 |
| Sample 42 | PD and insomnia | Male | 73 | 2 | 3 | 49 | 337.5 |
| Sample 43 | PD and insomnia | Female | 75 | 7 | 3 | 51 | 412.5 |
| Sample 44 | PD and insomnia | Male | 65 | 5 | 4 | 24 | 500 |
| Sample 45 | PD and insomnia | Female | 61 | 8 | 3 | 70 | 925 |
| Sample 46 | PD and insomnia | Female | 74 | 10 | 4 | 50 | 825 |
| Sample 47 | PD and insomnia | Female | 60 | 10 | 2 | 21 | 562.5 |
| Sample 48 | PD and insomnia | Female | 57 | 2 | 2 | 30 | 387.5 |
| Sample 49 | PD and insomnia | Male | 84 | 10 | 3 | 44 | 862.5 |
| Sample 50 | PD and insomnia | Female | 64 | 4 | 2.5 | 32 | 425 |
| Sample 51 | PD and insomnia | Male | 60 | 1 | 2 | 21 | 237.5 |
| Sample 52 | PD and insomnia | Male | 71 | 3 | 2 | 16 | 525 |
| Sample 53 | PD and insomnia | Female | 76 | 5 | 3 | 54 | 330 |
| Sample 54 | PD and insomnia | Female | 62 | 1 | 2 | 11 | 412.5 |
| Sample 55 | PD and RBD | Female | 51 | 1 | 2 | 18 | 375 |
| Sample 56 | PD and RBD | Female | 69 | 10 | 1 | 15 | 500 |
| Sample 57 | PD and RBD | Male | 66 | 2 | 2 | 27 | 462.5 |
| Sample 58 | PD and RBD | Female | 49 | 4 | 2 | 30 | 450 |
| Sample 59 | PD and RBD | Female | 73 | 20 | 2.5 | 49 | 545 |
| Sample 60 | PD and RBD | Female | 68 | 10 | 2.5 | 29 | 350 |
| Sample 61 | PD and RBD | Male | 55 | 2 | 2 | 14 | 50 |
| Sample 62 | PD and RBD | Male | 82 | 7 | 2.5 | 46 | 450 |
| Sample 63 | PD and RBD | Female | 58 | 3 | 2 | 28 | 225 |
| Sample 64 | PD and RBD | Male | 71 | 4 | 2.5 | 20 | 1062.5 |
| Sample 65 | PD and RBD | Male | 55 | 4 | 3 | 41 | 850 |
| Sample 66 | PD and RBD | Female | 62 | 1 | 2 | 16 | 237.5 |
| Sample 67 | PD and RBD | Male | 70 | 5 | 2.5 | 25 | 187.5 |
| Sample 68 | PD and RBD | Female | 73 | 5 | 4 | 30 | 387.5 |
| Sample 69 | PD and RBD | Male | 73 | 9 | 1 | 31 | 412.5 |
| Sample 70 | PD and RBD | Male | 79 | 7 | 3 | 59 | 375 |
| Sample 71 | PD and RBD | Male | 61 | 1 | 2 | 41 | 375 |
| Sample 72 | PD and RBD | Female | 84 | 1 | 2 | 18 | 337.5 |
| Sample 73 | PD and RBD | Female | 67 | 3 | 2.5 | 9 | 300 |
| Sample 74 | PD and RBD | Female | 68 | 0.5 | 3 | 58 | 687.5 |
| Sample 75 | PD and RBD | Female | 70 | 8 | 4 | 47 | 1025 |
| Sample 76 | PD and RBD | Female | 72 | 3 | 2.5 | 42 | 387.5 |
| Sample 77 | PD and RBD | Female | 69 | 1 | 2 | 54 | 487.5 |
| Sample 78 | PD and RBD | Female | 70 | 3 | 2 | 17 | 387.5 |
| Sample 79 | PD and RBD | Male | 76 | 7 | 3 | 35 | 375 |
| Sample 80 | PD and RBD | Female | 55 | 9 | 2.5 | 17 | 487.5 |
| Sample 81 | PD and RBD | Male | 64 | 20 | 3 | 55 | 987.5 |
| Sample 82 | PD and RBD | Male | 67 | 1 | 2 | 25 | 237.5 |
| Sample 83 | PD and RBD | Female | 70 | 3 | 2.5 | 18 | 425 |
| Sample 84 | PD and RBD | Male | 68 | 7 | 2 | 11 | 350 |
| Sample 85 | PD and RBD | Female | 68 | 10 | 3 | 46 | 487.5 |
| Sample 86 | PD and RBD | Male | 81 | 7 | 4 | 39 | 975 |
| Sample 87 | PD and RBD | Male | 77 | 7 | 4 | 38 | 575 |
| Sample 88 | PD and RBD | Female | 62 | 10 | 3 | 23 | 425 |
| Sample 89 | PD and RBD | Male | 65 | 9 | 2.5 | 30 | 337.5 |
| Sample 90 | PD and RBD | Male | 74 | 7 | 3 | 30 | 650 |
| Sample 91 | HC | Female | 52 | - | - | - | - |
| Sample 92 | HC | Female | 44 | - | - | - | - |
| Sample 93 | HC | Female | 44 | - | - | - | - |
| Sample 94 | HC | Male | 55 | - | - | - | - |
| Sample 95 | HC | Female | 56 | - | - | - | - |
| Sample 96 | HC | Female | 49 | - | - | - | - |
| Sample 97 | HC | Male | 54 | - | - | - | - |
| Sample 98 | HC | Female | 58 | - | - | - | - |
| Sample 99 | HC | Female | 45 | - | - | - | - |
| Sample 100 | HC | Female | 43 | - | - | - | - |
| Sample 101 | HC | Male | 52 | - | - | - | - |
| Sample 102 | HC | Male | 69 | - | - | - | - |
| Sample 103 | HC | Male | 51 | - | - | - | - |
| Sample 104 | HC | Male | 43 | - | - | - | - |
| Sample 105 | HC | Male | 52 | - | - | - | - |
| Sample 106 | HC | Male | 59 | - | - | - | - |
| Sample 107 | HC | Male | 67 | - | - | - | - |
| Sample 108 | HC | Male | 72 | - | - | - | - |
| Sample 109 | HC | Female | 67 | - | - | - | - |
| Sample 110 | HC | Female | 53 | - | - | - | - |
| Sample 111 | HC | Female | 52 | - | - | - | - |
